# The noncoding *MIR100HG* RNA enhances the autocrine function of transforming growth factor β signaling

**DOI:** 10.1038/s41388-021-01803-8

**Published:** 2021-05-04

**Authors:** Panagiotis Papoutsoglou, Dorival Mendes Rodrigues-Junior, Anita Morén, Andrew Bergman, Fredrik Pontén, Cédric Coulouarn, Laia Caja, Carl-Henrik Heldin, Aristidis Moustakas

**Affiliations:** 1 Department of Medical Biochemistry and Microbiology, Science for Life Laboratory, Box 582, Biomedical Center, Uppsala University, Uppsala, Sweden; 2 InInserm, Univ Rennes, UMR_S 1242, COSS (Chemistry, Oncogenesis Stress Signaling), Centre de Lutte contre le Cancer Eugène Marquis, Rennes, France; 3 Department of Immunology, Genetics and Pathology, Box 256, Rudbeck Laboratory, Uppsala University, Uppsala, Sweden

**Keywords:** Cancer, Oncogenes

## Abstract

Activation of the transforming growth factor β (TGFβ) pathway modulates the expression of genes involved in cell growth arrest, motility, and embryogenesis. An expression screen for long noncoding RNAs indicated that TGFβ induced *mir-100-let-7a-2-mir-125b-1* cluster host gene (*MIR100HG*) expression in diverse cancer types, thus confirming an earlier demonstration of TGFβ-mediated transcriptional induction of *MIR100HG* in pancreatic adenocarcinoma. *MIR100HG* depletion attenuated TGFβ signaling, expression of TGFβ-target genes, and TGFβ-mediated cell cycle arrest. Moreover, *MIR100HG* silencing inhibited both normal and cancer cell motility and enhanced the cytotoxicity of cytostatic drugs. *MIR100HG* overexpression had an inverse impact on TGFβ signaling responses. Screening for downstream effectors of *MIR100HG* identified the ligand TGFβ1. *MIR100HG* and *TGFB1* mRNA formed ribonucleoprotein complexes with the RNA-binding protein HuR, promoting TGFβ1 cytokine secretion. In addition, TGFβ regulated *let-7a-2–3p*, *miR-125b-5p*, and *miR-125b-1–3p* expression, all encoded by *MIR100HG* intron-3. Certain intron-3 miRNAs may be involved in TGFβ/SMAD-mediated responses (*let-7a-2–3p*) and others (*miR-100*, *miR-125b*) in resistance to cytotoxic drugs mediated by *MIR100HG*. In support of a model whereby TGFβ induces *MIR100HG*, which then enhances TGFβ1 secretion, analysis of human carcinomas showed that *MIR100HG* expression correlated with expression of *TGFB1* and its downstream extracellular target *TGFBI*. Thus, *MIR100HG* controls the magnitude of TGFβ signaling via TGFβ1 autoinduction and secretion in carcinomas.

## Introduction

Parts of the human genome are transcribed into noncoding RNAs (ncRNAs) that regulate gene expression, and have little or no protein-coding potential, such as microRNAs (miRNAs) [1] and long ncRNAs (lncRNAs) [2, 3]. Upon cleavage during splicing, either intronic or exonic regions of lncRNAs, called miRNA host genes, can give rise to miRNAs [2, 3], such as *miR-17–92a-1* cluster host gene (*MIR17HG*) [4], *miR-31* host gene (*MIR31HG*) [5], and *miR-100-let-7a-2-miR-125b-1* cluster host gene (*MIR100HG*) [6].

MiRNAs inhibit translation or induce degradation of mRNAs during development [7], and control proliferation and migration, which explains their deregulated expression in cancer [8]. *MiR17–92* favors cell cycle entry by targeting the cell cycle inhibitor p21^Cip1^ and retinoblastoma family proteins [8]. The *let-7* family targets cyclins and cyclin-dependent kinases (CDKs), while diverse cancers downregulate *let-7* expression [8]. *MiR-125b* targets CDK6 and other cell cycle regulators, acting as an antiproliferative miRNA [8]. MiRNAs may exert context-dependent actions in cancer, when viewed as units of signaling pathways.

One such pathway is transforming growth factor β (TGFβ) that signals via membrane receptors, which activate effector transcription factors (SMADs) and mitogen-activated protein kinases (MAPKs), to regulate target genes that control the cell cycle, migration, extracellular matrix remodeling, and epithelial–mesenchymal transition (EMT) [9]. Such target genes of TGFβ can be protein-coding or noncoding [10]. In normal or benign hyperplastic epithelial cells, TGFβ arrests the cell cycle and suppresses tumorigenesis by transcriptionally inducing the CDK inhibitors p15^Ink4b^, p21^Cip1^, and p27^Kip1^, and repressing the proto-oncogene *c-MYC* [11]. In advanced tumors, TGFβ promotes stemness, invasiveness, and metastasis [9]. For example, TGFβ induces *MIR100HG* expression in pancreatic tumors, generating *miR-100* and *miR-125b-1*, which act in a protumorigenic manner, and *let-7a-2*, which acts oppositely [6].

We screened for TGFβ-regulated lncRNAs in human keratinocytes and observed upregulation of *MIR100HG* [12], which was validated in diverse cell types. We then established a role of *MIR100HG* in TGFβ autoinduction, a central feature in TGFβ biology, especially in the context of cancer.

## Results

### TGFβ receptor-SMAD signaling induces *MIR100HG* expression in diverse normal and cancer cells

**Fig. 1:**
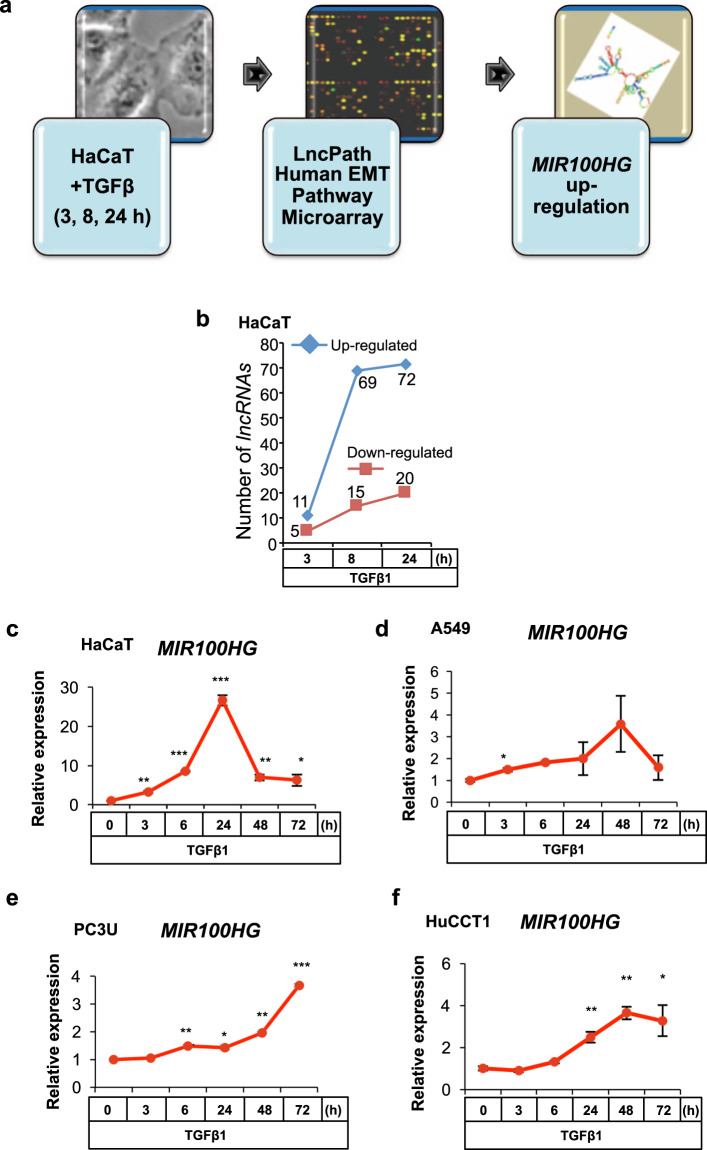
*MIR100HG* is induced by TGFβ. **a** Schematic outline of the experimental design to identify TGFβ-regulated lncRNAs. **b** Total numbers of lncRNA genes regulated by TGFβ1 in a time-course experiment, identified by microarray analysis. **c**–**f** Real-time RT-qPCR for determination of *MIR100HG* expression in HaCaT (**c**), A549 (**d**), PC3U (**e**), and HuCCT1 (**f**) cells in response to TGFβ1 treatment for the indicated time periods. Gene expression is normalized relative to the housekeeping genes *HPRT1* (**c**–**e**) or *TBP* (**f**). Error bars represent standard deviation from three different experiments (*P < 0.05, **P < 0.01, ***P < 0.001).

We searched for lncRNAs whose expression is regulated by the TGFβ pathway [12]. Microarray analysis in human HaCaT keratinocytes stimulated with TGFβ1 for early, intermediate, or long time periods, detected several up- or downregulated lncRNA genes (Fig. 1a, b). The screen was completed by selecting 23 TGFβ-regulated lncRNAs, silencing their expression by short-interfering (si) RNAs, and by monitoring CAGA_12_-luciferase activity, thus testing whether these lncRNAs affected TGFβ signaling [12]. The CAGA_12_-luciferase reporter monitors TGFβ signaling quantitatively by recruiting to its multimeric promoter the SMAD2/SMAD3/SMAD4 complex inducing the synthesis of luciferase transcripts.

*MIR100HG* lncRNA expression was upregulated by TGFβ stimulation with a peak of induction at 24 h (Fig. 1c), whereas its downregulation decreased CAGA_12_-luciferase reporter responsiveness (see “Results”) [12]. In addition to normal immortalized cells (Fig. 1c), TGFβ induced *MIR100HG* expression with cell-type-specific kinetics in A549 lung adenocarcinoma, PC3U prostate carcinoma, and HuCCT1 cholangiocarcinoma cells (Fig. 1d–f), and in pancreatic and lung adenocarcinoma cell line cohorts of the transcriptomic datasets GSE23952 [13] and GSE114761 [14] (Supplementary Fig. S1). These data agree with the first report of *MIR100HG* regulation by TGFβ in pancreatic tumors [6].

**Fig. 2:**
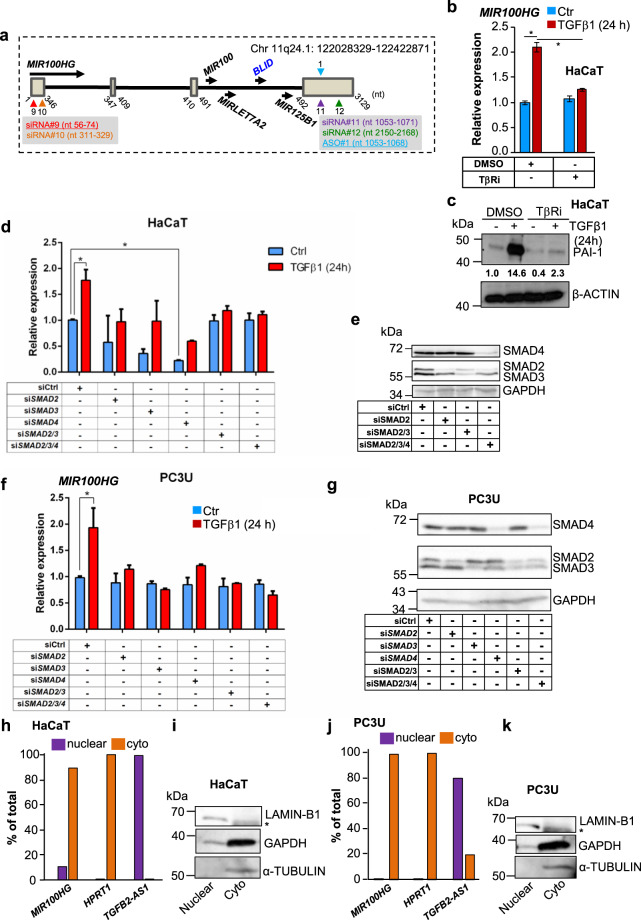
*MIR100HG* is induced by TGFβRI-SMAD signaling. **a** Schematic representation of the organization of the *MIR100HG* gene. Exons are shown as boxes and introns as lines. Arrows indicate the direction of transcription and the corresponding RNA (black) or protein-coding (blue) transcripts. The *MIR100HG* transcriptional unit coordinates on the H19 genome sequence (chromosome 11) are shown along with nucleotide (nt) numbering and coordinates of the four siRNAs and one ASO used in the study, marked by colored and numbered arrowheads, with underlines indicating those used in most experiments. **b** Real-time RT-qPCR for *MIR100HG* in HaCaT cells treated with TβRi (GW6604) in combination with TGFβ1 stimulation. Gene expression is normalized relative to the housekeeping gene *HPRT1*. Error bars represent standard deviation from three different experiments (*P < 0.05). **c** Immunoblotting for PAI-1 in HaCaT cells treated with TβRi (GW6604) in combination with TGFβ1 stimulation for 24 h. β-ACTIN was used as a loading control. Molecular mass (kDa) markers are indicated along with densitometric values of normalized band intensity. **d**, **f** Real-time RT-qPCR for determination of *MIR100HG* in HaCaT (**d**) or PC3U (**f**) cells transiently transfected with siRNAs targeting SMAD2, SMAD3, SMAD4, or combinations and treated or not with TGFβ1 for 24 h. Gene expression is normalized relative to the housekeeping gene *HPRT1*. Error bars represent standard deviation from three different experiments (*P < 0.05, **P < 0.01, ***P < 0.001). **e**, **g** Immunoblots corresponding to the experiment of panels (**d**, **f**) indicating the efficiency of SMAD protein silencing in specific combinations that cover all three SMADs examined. GAPDH was used as a loading control and molecular mass (kDa) markers are indicated. **h**–**k** Expression levels of *MIR100HG* lncRNA, *HPRT1* mRNA, and *TGFB2-AS1* lncRNA (which is known to be primarily nuclear) in nuclear and cytoplasmic fractions of HaCaT (**h**) or PC3U (**j**) lysates. Gene expression is normalized relative to the housekeeping gene *HPRT1*. These data show a representative experiment out of two. Corresponding immunoblot controls (**i**, **k**) verify the relative purity of cell fractions based on the nuclear (LAMIN-B1) and two cytoplasmic (GAPDH, α-TUBULIN) protein markers. Molecular mass (kDa) markers are indicated. A star indicates a nonspecific protein band recognized by the antibody.

*MIR100HG* spans 4 exons and 3 introns (NR_024430.2 transcript) on chromosome 11q24.1 and generates a 3129-nt-long spliced RNA (Fig. 2a). Intron-3 gives rise to *MIR-100*, a precursor to mature *miR-100-5p*/*-3p*, *MIRLET7A2*, generating *let-7a-5p*/*let-7a-2-3p*, *MIR125B1*, a precursor to mature *miR-125b-5p/miR-125b-1-3p*, and the BLID (BH3-like motif-containing cell death inducer) protein (Fig. 2a). Chemical inhibition of TGFβ receptor I (TGFβRI) kinase activity (TβRi: GW6604) in HaCaT cells, normalized TGFβ-induced *MIR100HG* expression to basal levels (Fig. 2b), as well as the established TGFβ-induced protein PAI-1 (plasminogen activator inhibitor-1, Fig. 2c). Silencing SMAD2, SMAD3, or SMAD4 alone or in combinations decreased TGFβ-induced *MIR100HG* expression in HaCaT, PC3U, A549, and HuCCT1 cells (Fig. 2d–g and Supplementary Fig. S2a–c). In the HuCCT1 model, an independent TβRi (LY2157299) blocked the induction of *MIR100HG* by TGFβ (Supplementary Fig. S2d). Chromatin immunoprecipitation (ChIP) revealed the association of SMAD2/3 to the *MIR100HG* promoter in HaCaT, PC3U, and A549 cells after stimulation with TGFβ (Supplementary Fig. S2e, f).

HaCaT and PC3U cell fractionation analysis confirmed that >90% of *MIR100HG* was cytoplasmic and <10% was nuclear (Fig. 2h–k). *HPRT1* mRNA was essentially cytoplasmic, as expected, and lncRNA *TGFB2-AS1* [12] was exclusively nuclear. Fraction purity was determined by analyzing the nuclear envelope protein lamin-B1 and the cytosolic proteins GAPDH and α-tubulin (Fig. 2i, k). Thus, several TGFβ-responsive cell models exhibit TGFβRI- and SMAD2/3/4-dependent transcriptional induction of *MIR100HG*, whose spliced product accumulates in the cytoplasm. In the following experiments, we studied *MIR100HG* expression and functional roles in PC3U, A549, and in nontumorigenic HaCaT cells, as these cell models showed robust responses of *MIR100HG* to TGFβ (Fig. 1), and represent cells of independent tissue origin.

### *MIR100HG* regulates diverse cell responses to TGFβ

**Fig. 3:**
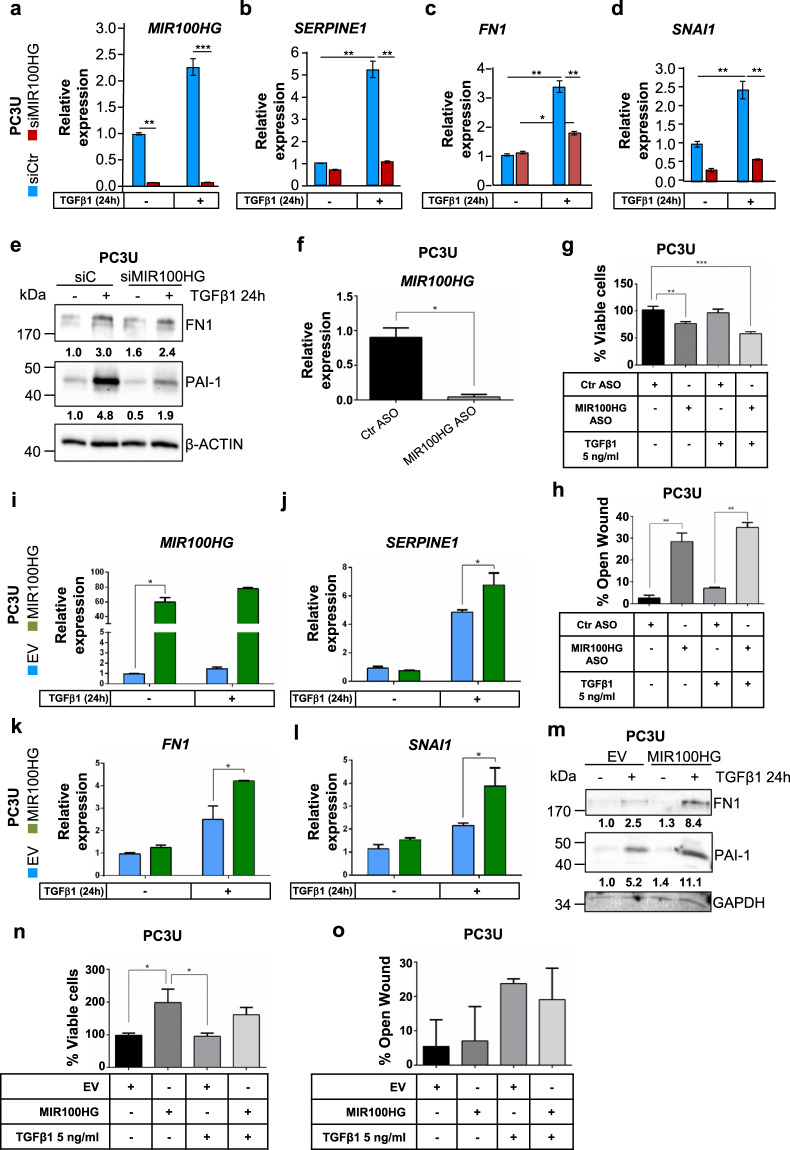
*MIR100HG* regulates TGFβ-signaling responses. **a**–**d** Real-time RT-qPCR for detection of *MIR100HG* (**a**), *SERPINE1* (**b**), *FN1* (**c**), and *SNAI1* (**d**) expression in PC3U cells transiently transfected with siMIR100HG#9 and stimulated with TGFβ1 or not for 24 h. Gene expression is normalized relative to the housekeeping gene *HPRT1*. Error bars represent standard deviation from three different experiments (*P < 0.05, **P < 0.01, ***P < 0.001). **e** Representative immunoblot out of three independent experiments for FN1 and PAI-1 in PC3U cells transiently transfected with control siRNA (siC) or siMIR100HG#9 and treated with TGFβ1 for 24 h. β-ACTIN was used as a loading control and molecular mass (kDa) markers are indicated along with densitometric values of normalized band intensity. **f** Real-time RT-qPCR for determination of *MIR100HG* expression in PC3U cells transiently transfected with anti-MIR100HG. Gene expression is normalized relative to the housekeeping gene *HPRT1*. Error bar represents the standard deviation from three different experiments (*P < 0.05). **g** Cell viability/proliferation assay with PC3U cells transiently transfected with negative control (Ctr) or specific ASO and treated or not with TGFβ1 for 24 h. Error bars represent standard deviation from three independent experiments (**P < 0.01, ***P < 0.001). **h** Cell migration assay with PC3U cells transiently transfected with negative control (Ctr) or specific ASO and treated or not with TGFβ1 for 24 h. Error bars represent standard deviation from three independent experiments (*P < 0.05, **P < 0.01). **i**–**l** Real-time RT-qPCR for detection of *MIR100HG* (i), which serves as a control for the experiments of panels (**m**–**o**), *SERPINE1* (**j**), *FN1* (**k**), and *SNAI1* (**l**) expression in PC3U cells transiently transfected with empty vector (EV) or pcDNA3-MIR100HG and stimulated with TGFβ1 or not for 24 h. Gene expression is normalized relative to the housekeeping gene *HPRT1*. Error bars represent standard deviation from three different experiments (*P < 0.05). **m** Representative immunoblot out of three independent experiments for expression of FN1 and PAI-1 in PC3U cells transiently transfected with empty vector (EV) or pcDNA3-MIR100HG and treated with TGFβ1 for 24 h. GAPDH was used as a loading control and molecular mass (kDa) markers are indicated along with densitometric values of normalized band intensity. **n** Cell viability/proliferation assay with PC3U cells transiently transfected with empty vector (EV) or pcDNA3-MIR100HG and treated or not with TGFβ1 for 24 h. Error bars represent standard deviation from three independent experiments (*P < 0.05). **o** Cell migration assay with PC3U cells transiently transfected with empty vector (EV) or *p*cDNA3-MIR100HG and treated or not with TGFβ1 for 24 h. Error bars represent standard deviation from three independent experiments. Lack of stars indicates a lack of statistical significance.

We investigated whether *MIR100HG* could affect expression of TGFβ target genes. Using a pool-of-4 siRNAs, two individual siRNAs targeting exons 1 and 4, and an independent antisense oligonucleotide (ASO) specific for exon 4 of *MIR100HG* (Fig. 2a), we identified those that silenced *MIR100HG* in HaCaT cells (Supplementary Fig. S3a), and chose siRNA#9 (exon 1) and ASO (exon 4) for the majority of subsequent experiments. Upon *MIR100HG* knockdown with siRNA#9 (Fig. 3a), inducibility of *SERPINE1* (encoding PAI-1), *FIBRONECTIN 1* (*FN1*), and *SNAI1/SNAIL* expression by TGFβ in PC3U cells was almost completely lost (Fig. 3b–d) (note retained weak inducibility of *FN1*, Fig. 3c), and basal *SNAI1* expression was suppressed (Fig. 3d), consistent with a role for *MIR100HG* to promote autocrine TGFβ signaling. *MIR100HG* silencing by siRNA#9 (Fig. 3a) reduced FN1 and PAI-1 protein levels (Fig. 3e). Although PC3U carcinoma cells do not exhibit physiological cell cycle arrest in response to TGFβ, upon *MIR100HG* silencing with ASO (Fig. 3f), their viability was reduced (Fig. 3g). Motility was strongly reduced in PC3U cells (Fig. 3h) upon *MIR100HG* silencing via ASO (Fig. 3f).

In nontumorigenic keratinocytes, *MIR100HG* silencing with siRNAPool-of-4 (Supplementary Fig. S3b) reduced induction of *SERPINE1* and *FN1* mRNAs (Supplementary Fig. S3c, d), and reduced FN1 and N-CAD protein levels, as well as phosphorylation of SMAD2 (Supplementary Fig. S3e), in response to TGFβ stimulation. In these experiments, silencing of SMAD3, SMAD4 (Fig. 2e), or TGFBR2 (not shown) dramatically blocked TGFβ-inducible *SERPINE1* and *FN1* expression (Supplementary Fig. S3c, d), indicating that the impact of *MIR100HG* silencing is in the same direction but not as robust as the impact of silencing central TGFβ signaling components (TGFBR2, SMAD3, and SMAD4). TGFβ-induced cell cycle arrest and cell number decrease were partially blocked (Supplementary Fig. S3e, f) after silencing *MIR100HG* with siRNA#9 or ASO (Supplementary Fig. S3a, d). Both assays also revealed a small but reproducible increase in HaCaT S-phase entry and viability upon *MIR100HG* silencing under basal conditions (Supplementary Fig. S3f, g). Motility was induced in HaCaT cells (Supplementary Fig. S3h) upon *MIR100HG* silencing via ASO (Supplementary Fig. S3a).

We cloned the human *MIR100HG* cDNA from immortalized mammary epithelial MCF10A cells (Supplementary Fig. S4a), and verified overexpression of *MIR100HG* in HaCaT (Supplementary Fig. S4b) and PC3U cells (Fig. 3i), which further enhanced basal and TGFβ-induced *SERPINE1*, *FN1*, and *SNAI1* mRNA levels and FN1 and PAI-1 protein levels in the PC3U cells (Fig. 3j–m), and the same proteins in HaCaT cells (Supplementary Fig. S4c). *MIR100HG* gain-of-function reduced viability of keratinocytes and cooperated with TGFβ by inducing a more robust growth inhibition (Supplementary Fig. S4d), whereas in PC3U cells, *MIR100HG* enhanced viability (Fig. 3n). Motility assays did not reveal significant differences upon *MIR100HG* overexpression in either cell model (Fig. 3o and Supplementary Fig. S4e). We conclude that *MIR100HG* silencing diminished, whereas *MIR100HG* overexpression strengthened TGFβ-mediated cell responses.

### Impact of *MIR100HG* on SMAD signaling and cytotoxicity

**Fig. 4:**
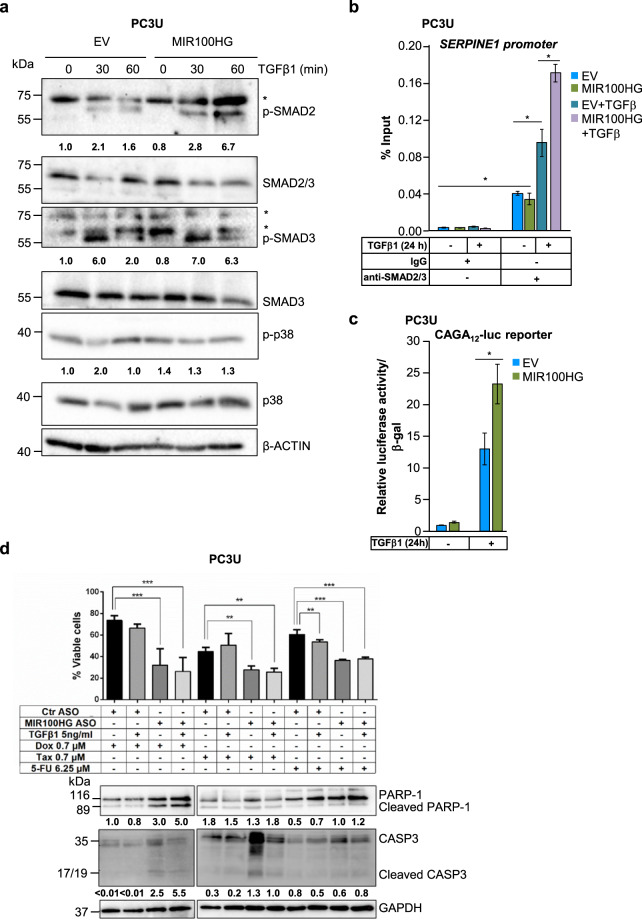
*MIR100HG* regulates TGFβ receptor signaling. **a** Representative immunoblots out of three independent experiments for expression of phosphorylated SMAD2 (p-SMAD2), SMAD2/3, phosphorylated SMAD3 (p-SMAD3), SMAD3, phosphorylated p38 (p-p38), and p38 in PC3U cells transiently transfected with empty vector (EV) or pcDNA3-MIR100HG (as shown in Fig. 3i) and treated with TGFβ1 for the indicated time periods. β-ACTIN was used as a loading control and molecular mass (kDa) markers are indicated along with densitometric values of normalized band intensity. Stars indicate nonspecific protein bands recognized by the antibodies. **b** ChIP-qPCR analysis for SMAD2/3 occupancy to the *SERPINE1/PAI-1* promoter in PC3U cells stimulated with TGFβ1 for 24 h or not, after transient transfection of empty vector (EV) or pcDNA3-MIR100HG (as shown in Fig. 3i). Control IgG immunoprecipitation data are also shown. Error bars represent standard deviation from three different experiments (*P < 0.05). **c** CAGA_12_-luciferase assay in PC3U cells transiently transfected with empty vector (EV) or pcDNA3-MIR100HG and in the presence or absence of TGFβ1 stimulation for 24 h (as shown in Fig. 3i). Error bars represent standard deviation from three different experiments (*P < 0.05). **d** Cell viability assay with PC3U cells transiently transfected with negative control (Ctr) or specific ASO (as shown in Fig. 3f) and treated with TGFβ1 in the absence or presence of the indicated concentrations of doxorubicin (Dox), taxol (Tax), or 5′-fluorouracil (5-FU) for 48 h. Error bars represent standard deviation from three independent experiments (**P < 0.01, ***P < 0.001).

We investigated whether *MIR100HG* regulates basal TGFβ signaling. Similar to HaCaT cells (Supplementary Fig. S3e), silencing *MIR100HG* with siRNA#9 in PC3U cells (Fig. 3a) decreased the well-established early phosphorylation of SMAD2, SMAD3, and p38 MAPK after 30 min of TGFβ stimulation (Supplementary Fig. S5a). Relative to the control, nonspecific ASO, which allowed proper time-dependent TGFβ signaling, anti-*MIR100HG* ASO (silencing efficiency shown in Fig. 3f), decreased the phosphorylated levels of SMAD2, SMAD3, and p38 after 30 and 60 min of TGFβ stimulation (Supplementary Fig. S5b). Conversely, *MIR100HG* overexpression (Fig. 3i) enhanced phosphorylation of SMAD2 and SMAD3 (Fig. 4a). SMAD2/SMAD3 signaling directly translates to target gene promoter/enhancer binding and regulation. Accordingly, *MIR100HG* overexpression approximately doubled SMAD2/3 recruitment to the *SERPINE1* promoter after TGFβ stimulation, determined by ChIP-qPCR in PC3U cells (Fig. 4b), and mirroring the impact *MIR100HG* overexpression has on CAGA_12_-luciferase activity (Fig. 4c). In a similar fashion, TGFβ-induced CAGA_12_-luciferase activity in keratinocytes was reduced upon *MIR100HG* silencing with the siRNApool to an extent comparable to TGFBR2 silencing (Supplementary Fig. S5c), and was further enhanced upon *MIR100HG* overexpression (Supplementary Fig. S5d). To explore deeper effects of *MIR100HG* on cell viability, we used the cytotoxic drugs doxorubicin, taxol, and 5-fluorouracil in combination with TGFβ stimulation and/or *MIR100HG* silencing (Fig. 4d). Drug cotreatment with TGFβ enhanced cytotoxicity only in the case of 5-fluorouracil; *MIR100HG* silencing reproducibly enhanced cytotoxicity by all drugs, as revealed by viability, cleaved PARP-1, and caspase-3 analysis (Fig. 4d). Thus, *MIR100HG* possibly acts at the level of TGFβ ligand and/or TGFβ receptors.

### *MIR100HG* regulates TGFβ1 expression via the RNA-binding protein HuR

**Fig. 5:**
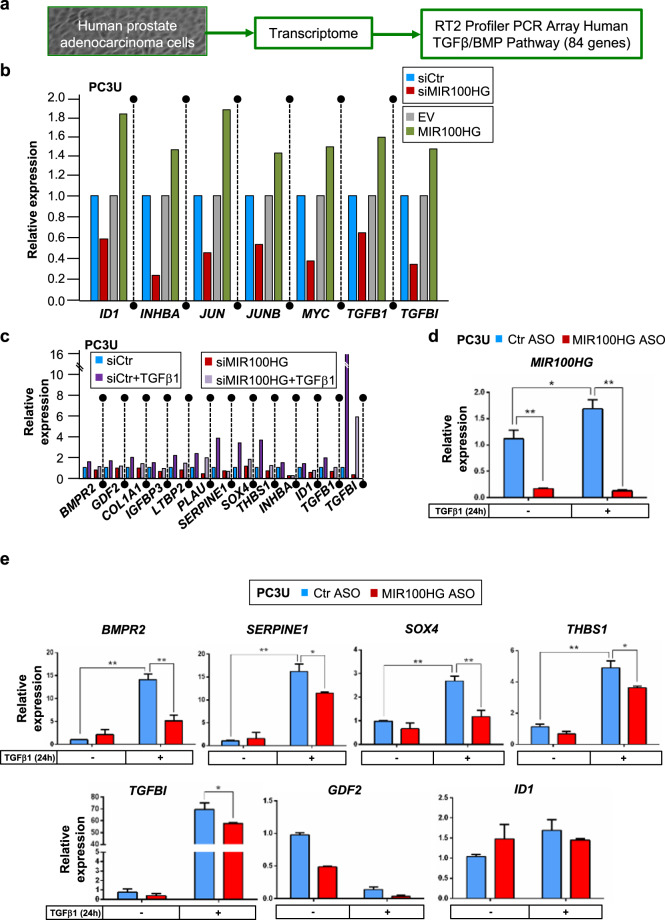
*MIR100HG* regulates *TGFB1* expression and many members of the fibrogenic program. **a** Schematic outline of the experimental design to identify TGFβ-signaling targets of *MIR100HG* action. **b** Gene expression data based on the RT^2^ profiler PCR array of the human TGFβ/BMP signaling pathway in PC3U cells transiently transfected with control siRNA (siCtrl) or siMIR100HG#9 (as shown in Fig. 3a) and with empty vector (EV) or pcDNA3-MIR100HG (as shown in Fig. 3i). Data from a single biological repeat are shown, highlighting genes whose expression was affected by both silencing and overexpression of *MIR100HG*. **c** Similar experiment as in panel (**b**) with the addition of TGFβ1 stimulation for 24 h in PC3U cells. **d**, **e** Validation of PCR array analysis using real-time RT-qPCR for detection of *MIR100HG*, *BMPR2*, *SERPINE1*, *SOX4*, *THBS1*, *TGFBI*, *GDF2*, and *ID1* expression in PC3U cells transiently transfected with negative control or specific ASO and stimulated or not with TGFβ1 for 24 h. Gene expression is normalized relative to the housekeeping gene *HPRT1*. Error bars represent standard deviation from three different experiments (*P < 0.05, **P < 0.01).

We examined the impact of *MIR100HG* on expression of TGFβ family signaling genes using a microarray platform that provided strong indications but did not allow reproducibility assays for technical reasons (Fig. 5a). Many of the 84 investigated transcripts were readily expressed in PC3U cells (Supplementary Fig. S6). The ligands *TGFB1* and *INHBA*, their downstream effector of cell adhesion and secreted glycoprotein *TGFBI*, and downstream transcription factors *ID1*, *MYC*, *JUN*, and *JUNB*, were downregulated upon *MIR100HG* silencing (siRNA#9, Fig. 3a) and upregulated upon *MIR100HG* overexpression (Fig. 5b, single biological repeat). After TGFβ stimulation, *TGFB1*, *INHBA,* and its downstream target genes *IGFBP3*, *SERPINE1*, *SOX4*, *THBS1*, *ID1*, and *TGFBI* were most robustly inhibited after silencing *MIR100HG* (Fig. 5c, single biological repeat, siRNA#9). Independent RT-qPCR assays confirmed in multiple repeats that *MIR100HG* silencing (Fig. 5d, via ASO) resulted in a relative decrease of *TGFB1* (see next section), *BMPR2*, *SERPINE1*, *SOX4*, *THBS1, and TGFBI;* expression of *GDF2* or *ID1* also decreased, but not significantly (Fig. 5e), in agreement with lack of significant regulation by *MIR100HG* in the single-microarray assay (Fig. 5c). Many of the analyzed genes mediate a conserved *TGFB1* autoinduction mechanism that responds to TGFβ signaling.

**Fig. 6:**
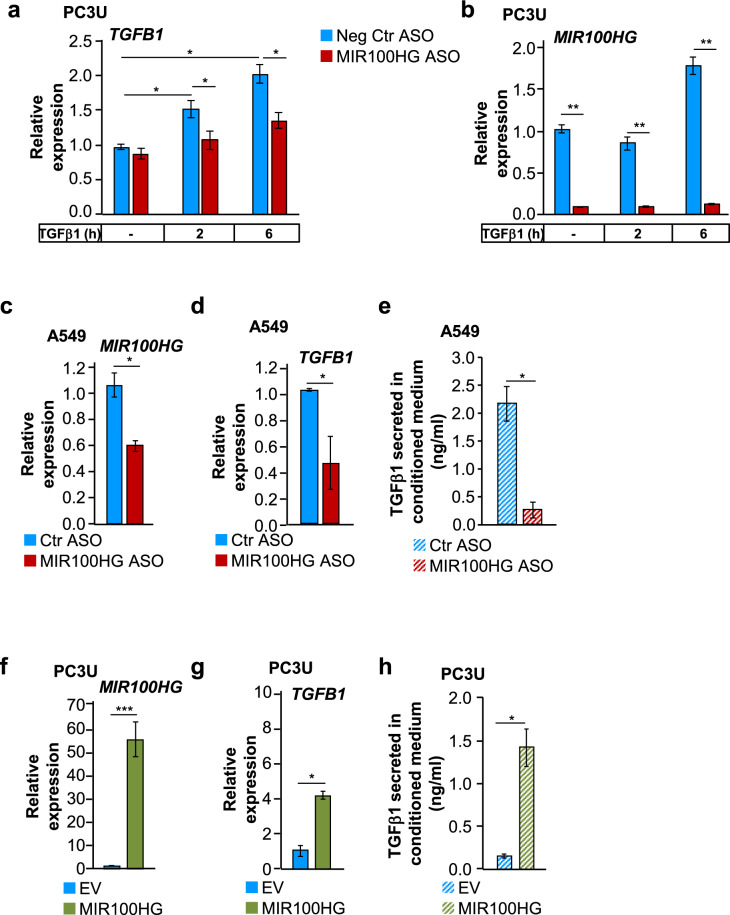
TGFβ1 synthesis and secretion is induced by *MIR100HG* downstream of TGFβ signaling. **a**, **b** Real-time RT-qPCR for detection of *TGFB1* (**a**) and *MIR100HG* (**b**) expression in PC3U cells transiently transfected with negative control or MIR100HG-specific ASO and stimulated with TGFβ1 or not for the indicated time periods. Gene expression is normalized relative to the housekeeping gene *HPRT1*. Error bars represent standard deviation from three different experiments (*P < 0.05, **P < 0.01). **c**–**h** Detection of *MIR100HG* (**c**, **f**), *TGFB1* mRNA (**d**, **g**) expression by real-time RT-qPCR, and corresponding secreted mature TGFβ1 ligand detected by ELISA in the conditioned medium (**e**, **h**) of A549 (**c**–**e**) and PC3U (**f**–**h**) cells transiently transfected with negative control or *MIR100HG*-specific ASO in the absence of TGFβ stimulation. Gene expression is normalized relative to the housekeeping gene *HPRT1*. Error bars represent standard deviation from three different experiments (*P < 0.05, ***P < 0.001).

We corroborated the impact of *MIR100HG* on TGFβ1 expression (Fig. 6). The anti-*MIR100HG* ASO suppressed *TGFB1* mRNA expression in PC3U and A549 cells (Fig. 6a–d) and suppressed secreted TGFβ1 protein in A549-conditioned medium (Fig. 6e). *MIR100HG* overexpression enhanced both *TGFB1* mRNA and protein secretion in PC3U cells (Fig. 6f–h). The impact *MIR100HG* had on secreted TGFβ1 suggested post-transcriptional action.

**Fig. 7:**
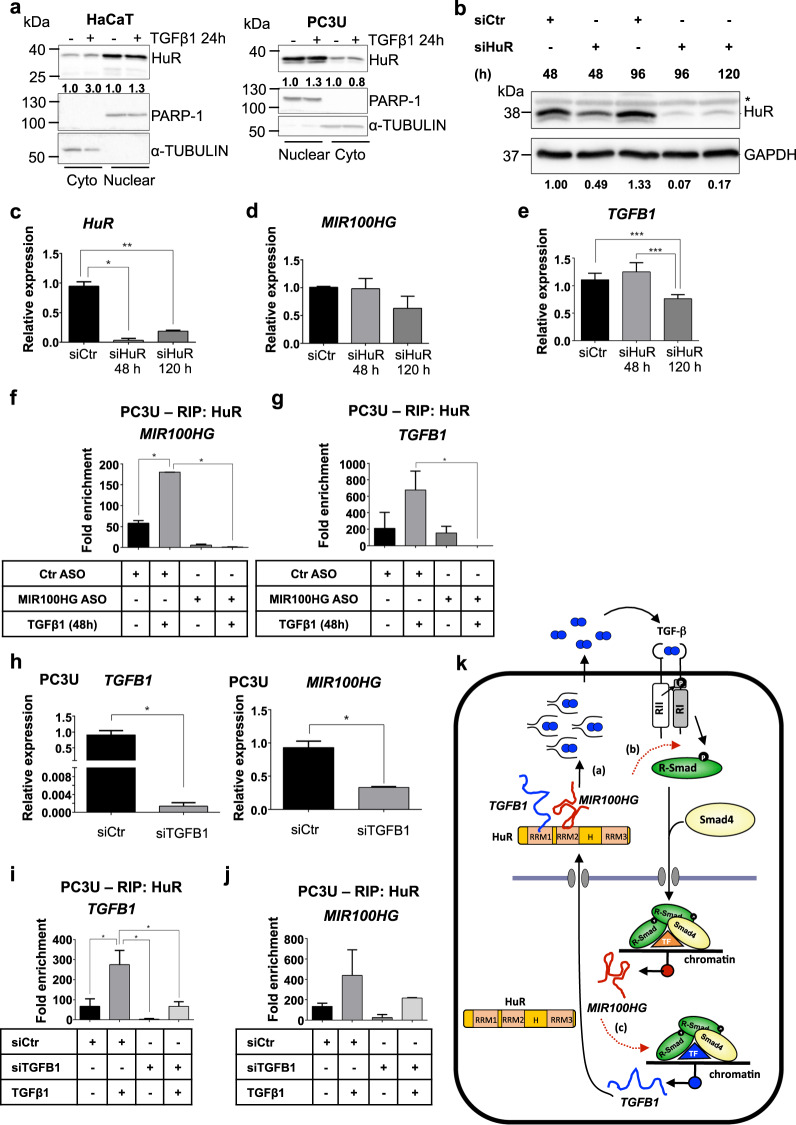
*MIR100HG* engages HuR to regulate TGFβ1. **a** Representative immunoblots out of two independent experiments for expression of HuR in HaCaT (left) and PC3U (right) cells treated with TGFβ1 for 24 h. PARP-1 (nuclear) and α-TUBULIN (cytoplasmic) were used as fractionation controls and molecular mass (kDa) markers are indicated along with densitometric values of normalized band intensity. **b**–**e** Representative immunoblot (**b**) out of three independent experiments for expression of HuR, and corresponding real-time RT-qPCR for detection of *HuR* (**c**), *MIR100HG* (**d**), and *TGFB1* (**e**) mRNA expression in PC3U cells transiently transfected with negative control or specific siRNA for the indicated time periods. GAPDH was used as loading control and molecular mass (kDa) markers are indicated. A star indicates nonspecific protein bands recognized by the antibody. Gene expression is normalized relative to the housekeeping gene *HPRT1*. Error bars represent standard deviation from three different experiments (*P < 0.05, **P < 0.01, ***P < 0.001). **f**, **g** RIP analysis in PC3U cells transiently transfected with negative control or *MIR100HG*-specific ASO and stimulated with TGFβ1 or not for 48 h. Fold enrichment of the HuR-specific RIP relative to the IgG control is reported for *MIR100HG* (**f**) and *TGFB1* (**g**) RNAs. Error bars represent standard deviation from three different experiments (*P < 0.05). **h** Real-time RT-qPCR for detection of *TGFB1* and *MIR100HG* expression in PC3U cells transiently transfected with negative control or *TGFB1*-specific siRNA and treated with TGFβ1 for 48 h. Gene expression is normalized relative to the housekeeping gene *HPRT1*. Error bars represent standard deviation from three different experiments (*P < 0.05). **i**, **j** RIP analysis in PC3U cells transiently transfected with negative control or *TGFB1*-specific siRNA and stimulated with TGFβ1 or not for 48 h. Fold enrichment of the HuR-specific RIP relative to the IgG control is reported for *TGFB1* (**i**) and *MIR100HG* (**j**) RNAs. Error bars represent standard deviation from three different experiments (*P < 0.05). **k** Diagrammatic scheme of TGFβ signaling regulating the downstream target genes *MIR100HG* and *TGFB1*, mediated by the two TGFβ receptor kinases and SMAD complexes together with gene-specific transcription factors (TF, note color differentiation based on gene specificity), leading to transcriptional induction of *MIR100HG* and *TGFB1*. Two possible (dashed red arrows) and one confirmed mechanism of action of *MIR100HG* are illustrated: (a) cytoplasmic *MIR100HG* associates with HuR in the cytoplasm (HuR domains are highlighted) and causes stabilization and accumulation of *TGFB1* mRNA, which leads to enhanced synthesis of latent and mature secreted TGFβ1 (dimeric circles—mature TGFβ1—with twinkled lines—N-terminal pro-domain) that further stimulates the pathway in an autocrine manner. The two RNAs are shown to interact with distinct HuR RRM domains (not proven) and for completion, nuclear HuR is also illustrated. (b) Cytoplasmic *MIR100HG* promotes or stabilizes TGFβ receptor-SMAD complexes that prolong signaling. (c) A transcriptional mechanism in which nuclear *MIR100HG* enhances SMAD-mediated transcription of the *TGFB1* gene.

To investigate post-transcriptional mechanisms, we focused on the RNA-binding protein human antigen R (HuR) [15, 16], a nuclear protein that shuttles to the cytoplasm, associates with AU-rich sequences at the 3′ untranslated region of mRNAs, including *TGFB1* mRNA, causing stabilization and enhancing their translation [17, 18]. Furthermore, *MIR100HG* can associate with HuR, facilitating interactions between HuR and mRNAs [19]. Stimulating or not keratinocytes and PC3U cells with TGFβ confirmed that HuR partitioned mainly in the nucleus and exhibited a substantial cytoplasmic pool (Fig. 7a). Silencing HuR using two out of four independent siRNAs (Fig. 7b, c) did not significantly affect steady-state *MIR100HG* levels, but decreased *TGFB1* mRNA levels (Fig. 7d, e), and weakly but not-significantly decreased secreted TGFβ1 protein (Supplementary Fig. S7).

RNA immunoprecipitation (RIP) assays in PC3U cells transfected with control or anti-*MIR100HG* ASO, exhibiting robust *MIR100HG* silencing and corresponding *TGFB1* mRNA reduction (Supplementary Fig. S8a, b), demonstrated HuR immunocomplexes with high amounts of *MIR100HG* (relative to control IgG), and measurable amounts of *TGFB1* mRNA (Supplementary Fig. S8c, d). Calculating fold enrichment of each RNA in HuR immunocomplexes relative to nonspecific IgG, showed that TGFβ stimulation significantly enhanced HuR-*MIR100HG* association, and *MIR100HG* silencing eliminated these complexes, as expected (Fig. 7f). Confirming our hypothesis, the HuR-*TGFB1* complexes lost a large portion of bound *TGFB1* mRNA upon *MIR100HG* silencing (Fig. 7g and Supplementary Fig. S8d). As an additional control, *TGFB1* was silenced, decreasing basal *MIR100HG* levels as expected (Fig. 7h), and after TGFβ stimulation, causing observable but weaker *MIR100HG* decrease (Supplementary Fig. S8e, f). RIP demonstrated again HuR-*TGFB1* association, which decreased, as expected, after *TGFB1* silencing (Fig. 7i and Supplementary Fig. S8g), and HuR-*MIR100HG* complexes showed a decreasing but not statistically significant trend (Fig. 7j and Supplementary Fig. S8h). The data suggest that *MIR100HG* facilitates the formation of HuR-*TGFB1* ribonucleoprotein complexes (Fig. 7k).

### Regulation of miRNA expression from the *MIR100HG* intron-3

Since unspliced *MIR100HG* can be processed into miRNAs (Fig. 2a), signaling inputs that induce spliced *MIR100HG* should regulate *MIR100HG*-derived miRNAs, as they share the same transcriptional promoter. *Pre-miR-100* showed time-dependent downregulation in HaCaT cells and stable levels in PC3U cells in response to TGFβ stimulation (Supplementary Figs. S9a, S10a). Mature *miR-100-5p* showed a late-time but not significant trend of upregulation in both cell lines (Supplementary Figs. S9b, S10b), and also increased weakly in A549 cells after 24-h stimulation (Supplementary Fig. S11a). *MiR-100-3p* expression was undetectable in all cell lines. *MiR-361-5p* expression, used as a reference, remained unchanged in response to TGFβ (Supplementary Fig. S11b). *Pre-miR-125b* showed early induction by TGFβ in HaCaT cells and early downregulation in PC3U cells (Supplementary Figs. S9c, S10c). The corresponding mature miRNAs showed (not significant) trends for upregulation in HaCaT (Supplementary Fig. S9d, e), early downregulation but only for *miR-125b-3p* in PC3U cells (Supplementary Fig. S10d, e), and significant upregulation at 24 h in A549 cells (Supplementary Fig. S11c, d). *Pre-miR-let7a-2* showed early upregulation in HaCaT cells and a corresponding (but not significant) trend in PC3U cells (Supplementary Figs. S9f, S10f). Mature *let-7a-2* miRNAs showed corresponding trends of upregulation in HaCaT and PC3U cells (Supplementary Figs. S9g, h, S10g, h), whereas significant upregulation only for *let-7a-2–3p* was observed in A549 cells (Supplementary Fig. S11e, f). Thus, TGFβ can regulate expression of some of the miRNAs of the *MIR100HG* intron-3, in a cell-type- and time-dependent manner; however, the kinetics of regulation of the miRNAs do not match those of spliced *MIR100HG* and the regulation is highly variable.

In order to test whether spliced *MIR100HG* affects intron-3 miRNA biogenesis, we silenced spliced *MIR100HG* (using siRNApool, Supplementary Fig. S12a) in HaCaT cells, and detected no impact on basal expression of the five miRNAs or on TGFβ-induced levels of *miR-125b-1–3p* and *let-7a-2–3p* (Supplementary Fig. S12). In PC3U cells though, *MIR100HG* silencing (via ASO, Supplementary Fig. S3a) had no effect on *pre-miR-100*, but resulted in significant downregulation of mature *miR-100-5p*, *pre-miR-125b*, and *miR-125b-1–3p/5p* (Supplementary Fig. S13). The cumulative data suggest that spliced *MIR100HG* functions in parallel and possibly independently from the intron-3 miRNAs, however, a clear impact on mature *miR-100-5p*, *miR-125b-5p*, and *miR-125b-1–3p* expression is worth considering.

### *Let-7a-2–3p* regulates TGFβ-target genes and epithelial cytostasis

We also asked whether intron-3 miRNAs could affect TGFβ signaling output. We overexpressed chemically stabilized mimics of mature miRNAs whose expression was regulated by TGFβ (*let-7a-2–3p*, *miR-125b-1–3p*, and *miR-125b-5p*) or negative control random-sequence miRNA mimic (NCm) in HaCaT cells. *Let-7a-2–3p* mimic but not *miR-125b-1–3p* mimic or *miR-125b-5p* mimic potentiated TGFβ-induced CAGA_12_-luciferase activity (Supplementary Fig. S14a). Cotransfecting all three miRNA mimics (*let-7a-2–3p*, *miR-125b-5p*, and *miR-125b-1–3p*) enhanced TGFβ-induced CAGA_12_-luciferase activity (Supplementary Fig. S14b), to a similar degree as achieved by single *let-7a-2–3p* mimic, failing to demonstrate additive effects.

Furthermore, ectopic *let-7a-2–3p* mimic, enforced significant cell cycle arrest, whereas *miR-125b-5p* mimic and *miR-125b-1–3p* mimic showed a trend but not significant effect (Supplementary Fig. S14c). TGFβ stimulation further enhanced the cytostatic response (Supplementary Fig. S14c). We also monitored *let-7a-2–3p* effects on expression of CDK inhibitors downstream of TGFβ signaling, observing enhancement of *CDKN2B/p15*^*Ink4b*^ expression induced by TGFβ stimulation, and strong upregulation of *CDKN1A/p21*^*Cip1*^ expression in control and TGFβ-treated cells (Supplementary Fig. S14d, e).

Analyzing genes of the TGFβ fibrogenic program, *let-7a-2–3p* mimic enhanced TGFβ-induced *SERPINE1* and *FN1* expression (Supplementary Fig. S14f). Inhibiting endogenous *let-7a-2–3p* but not *let-7a-5p* (produced from the 5′-arm of the *pre-let-7a-2*) or the reference gene *miR-361-5p*, weakly attenuated TGFβ-induced *SERPINE1* and *FN1* levels, PAI-1 and N-CAD protein levels, without clear effect on FN1 protein, and phospho-SMAD2 levels in HaCaT cells (Supplementary Fig. S15).

We attempted to identify potential targets of *let-7a-2–3p* using the DIANA online suite [20]. After removing 54 common predicted targets of *let-7a-5p* and *let-7a-2–3p*, we observed several highly significant pathways encompassing the target mRNAs, including glycosaminoglycan biosynthetic enzymes, Wnt signaling components, and histone methyltransferases (Supplementary Fig. S16a, b). Parallel querying of the KEGG database for all *let-7a-2* target mRNAs revealed additional and diverse functional categories (Supplementary Fig. S16c–e). Focusing on the *let-7a-2–3p* targets, Wnt and estrogen signaling are known to crosstalk with TGFβ and provide relevant points for analyzing the role of *let-7a-2* in the context of cancer biology. Collectively, intron-3 miRNAs can, to some extent, contribute to TGFβ responses, whereas the impact of *MIR100HG* was more robust and general.

### *MIR100HG* and TGFβ1 expression profiles in diverse tumors

**Fig. 8:**
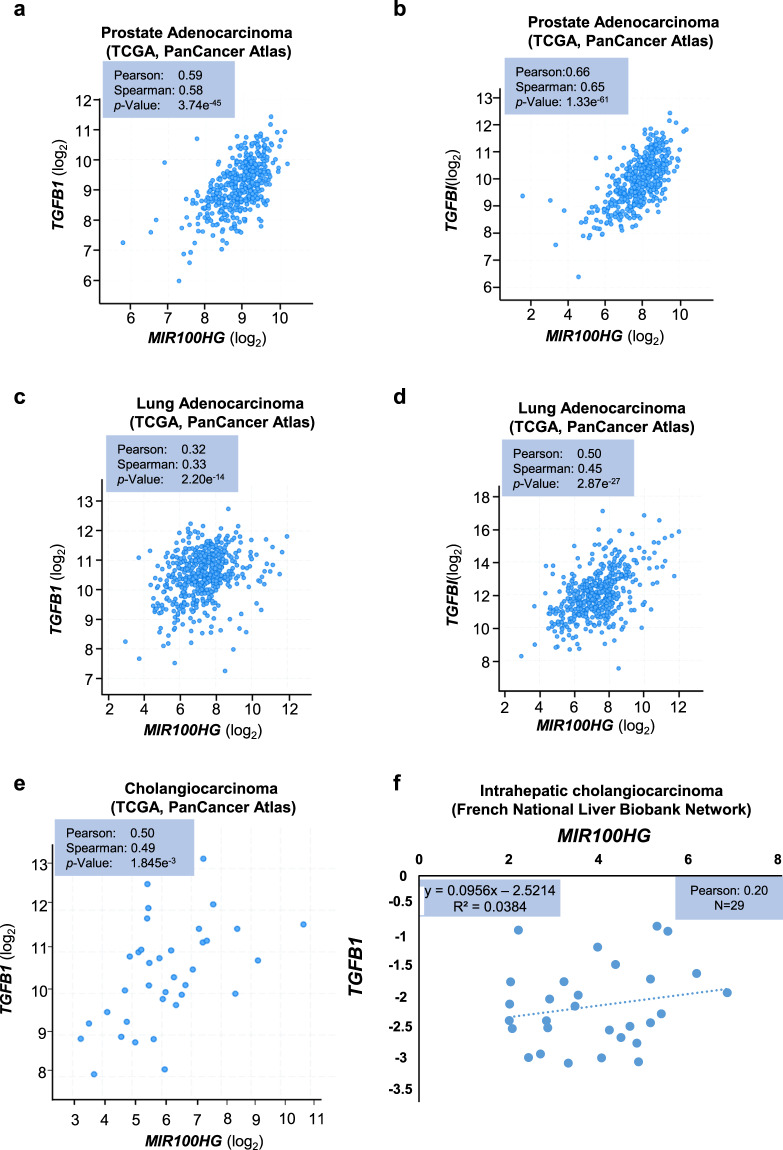
*MIR100HG* and *TGFB1* expression analysis in human cancers. **a**–**e** Expression correlation of *TGFB1* and *MIR100HG* (**a**, **c**, **e**) or *TGFBI* and *MIR100HG* (**b**, **d**) in prostate adenocarcinoma (**a**, **b**), lung adenocarcinoma (**c**, **d**), and cholangiocarcinoma (**e**) patients enlisted in the PanCancer Atlas. Expression values are reported in a natural logarithmic scale and Pearson and Spearman correlation values are indicated along with the corresponding *P* values. **f** Expression correlation of *TGFB1* and *MIR100HG* in 29 samples from intrahepatic cholangiocarcinoma patients included in the French national liver biobank network.

Since TGFβ induces *MIR100HG*, which enhances TGFβ signaling in normal and carcinoma cells, we examined *MIR100HG* expression in cancer patients. Querying the PanCancer Atlas expression data from ~10,000 patients [21], revealed that *MIR100HG* expression was significantly higher in the lung (where A549 cells belong) and prostate (where PC3U cells belong) carcinoma relative to other tumors (Supplementary Fig. S17a). A PanCancer Atlas cohort of 494 prostate adenocarcinoma patients [21] revealed a positive correlation between *MIR100HG* and *TGFB1* expression (Fig. 8a), and even stronger correlation between *MIR100HG* and *TGFBI* expression (Fig. 8b). Significant correlations with weaker coefficients were observed in 566 lung adenocarcinoma samples and in 36 cholangiocarcinoma samples of the PanCancer Atlas (Fig. 8c–e), the latter being confirmed with data from an independent cholangiocarcinoma cohort representing 29 patients (Fig. 8f). Using in situ hybridization on a cancer patient tissue microarray, we detected distinct cytoplasmic *MIR100HG* signals in lung adenocarcinoma, higher expression in malignant melanoma, and even higher in glioma specimen (Supplementary Fig. S17b), reflecting the findings of the PanCancer Atlas cohorts (Supplementary Fig. S17a).

Since TGFβ signaling is known to play both tumor suppressor and protumorigenic roles dependent on the cancer type and stage [9], we analyzed contributions of *MIR100HG* and *TGFB1* to overall survival of patients with different tumors. Thus, using OncoLnc [22], Kaplan–Meier analysis revealed that in lung adenocarcinoma and cutaneous melanoma, high expression of both *MIR100HG* and *TGFB1* was linked to long patient survival (Supplementary Fig. S18a–d), suggesting their tumor-suppressive role. Conversely, high *MIR100HG* and *TGFB1* expression correlated with poor survival outcome in stomach adenocarcinoma (Supplementary Fig. S18e, f). The cancer database analyses must be considered with caution, yet they suggest that *MIR100HG* and *TGFB1* present similar predictive values in the prognosis of certain cancers.

## Discussion

We here establish that TGFβ upregulates spliced *MIR100HG*, which positively regulates TGFβ responses in several normal and cancer cell types (Figs. 1–4). Signaling via the TGFβRI and SMAD proteins (Fig. 2), but possibly additional mediators (e.g. MAPK) participate in this regulation, as previously established for pancreatic carcinomas [6]. Mechanistically, *MIR100HG* facilitates the RNA-binding protein HuR to form complexes with the *TGFB1* mRNA, thus stabilizing the mRNA and enhancing autocrine TGFβ1 production and autogenous responses (Fig. 7k). Our observations are compatible with recent findings that demonstrated a function of *MIR100HG* as a HuR protein facilitator [19], and extend this model in the context of TGFβ cancer biology. The 3′ untranslated region of the *TGFB1* mRNA contains AU-rich sequences recognized by HuR [18]. Furthermore, HuR-mediated stabilization of *TGFB1* mRNA can sustain TGFβ signaling in cardiac fibroblasts during fibrosis [17]. Combined RIP and cell fractionation analyses confirm this model in the context of cancer cell biology (Fig. 7). In fibroblasts, TGFβ signaling was shown to induce translocation of nuclear HuR to the cytoplasm where association with mRNA seems to take place [17]. In normal epithelial and carcinoma cells, we observed weak mobilization of nuclear HuR to the cytoplasm in response to TGFβ (Fig. 7a). Yet, we find more convincing the fact that *MIR100HG* accumulates in the cytoplasm of all cell types examined (Fig. 2h–k), suggesting that *TGFB1* mRNA stabilization depends on cytoplasmic accumulation of *MIR100HG*. Interestingly, RIP experiments reproducibly showed that TGFβ signaling enhanced association between HuR, *MIR100HG*, and *TGFB1* mRNA (Fig. 7g–i), suggesting that TGFβ, in addition to promoting transcription from the *MIR100HG* and *TGFB1* genes, also regulates cytoplasmic RNA-protein assembly (Fig. 7k). HuR encompasses three RNA-recognition motifs (RRM1–3, Fig. 7k), one of which facilitates HuR dimerization [23]. We envision a mechanism whereby *MIR100HG* binding to one RRM, facilitates the association of *TGFB1* mRNA with a second RRM in dimeric HuR (Fig. 7k).

Beyond the above mechanism, additional processes explaining oncogenic roles of *MIR100HG* have been reported. In acute megakaryoblastic leukemia, silencing of *MIR100HG* impaired cell proliferation [24]. In osteosarcoma cells, *MIR100HG* expression peaked during early G1 cell cycle phase, and *MIR100HG* silencing caused cell cycle arrest [19]. In osteosarcoma cells, *MIR100HG* promotes proliferation by interacting with the EZH2 protein of the polycomb repressor complex-2, causing repression of *LATS1/2*, mediators of Hippo signaling [25]. In breast cancer cells, triple-helical formation between *MIR100HG* and *p27*^*Kip1*^ DNA suppresses *p27*^*Kip1*^ and promotes proliferation [26].

As TGFβ signaling instructs the *MIR100HG* promoter, it may also affect the maturation of miRNAs derived from *MIR100HG* intron-3. TGFβ regulated expression of some but not all *MIR100HG* miRNAs with diverse kinetic profiles among different carcinoma cells (Supplementary Figs. S9 and S10). Selective regulation of miRNA maturation could be mediated by the direct association of SMAD3 with specific pre-miRNAs and the enzyme DROSHA [27]. However, *MIR100HG*-miRNAs were not among those processed via the TGFβ/SMAD3/DROSHA-specific mechanism [27]. Furthermore, TGFβ induced expression of *miR-100* and *miR-125b*, but not of *let-7a-2* in pancreatic adenocarcinoma cells [6]. The distinct *MIR100HG-*miRNAs induced by TGFβ in keratinocytes and diverse carcinoma cells (this study) or in pancreatic adenocarcinoma [6] may reflect different physiological outcomes mediated by TGFβ. We suggest that induction of the antitumorigenic *let-7a-2-3p* by TGFβ enhances its antiproliferative effect (Supplementary Fig. S14c). On the other hand, in pancreatic adenocarcinoma, TGFβ frequently loses its antiproliferative power and instead promotes EMT and stemness [28], including enhanced expression of *miR-100* and *miR-125b*, which promote EMT [6]. Additional studies highlight protumorigenic actions for *miR-100* and *miR-125b;* chemotherapy-resistant colorectal cancer cells exhibit high *MIR100HG*, *miR-100*, and *miR-125b* expression, the miRNAs enhancing Wnt signaling [29]. In prostate carcinoma PC3U cells, silencing of spliced *MIR100HG* downregulated expression of mature *miR-100* and *miR-125b* (Supplementary Fig. S14c–e). This may explain why silencing *MIR100HG* sensitized the PC3U cells to cytotoxic drugs (Fig. 4d). Furthermore, *miR-100* induces mammary EMT by downregulating the chromatin remodeling factor SMARCA5 [30]. We observed that silencing *MIR100HG* reduced TGFβ responses and overexpression of *MIR100HG*, lacking introns and therefore unable to influence intron-3 miRNA biosynthesis (data not shown), resulted in enhanced TGFβ responses (Figs. 3 and 4). Thus, *MIR100HG* has functions beyond hosting miRNAs in one if its introns.

The link established here between *MIR100HG* and *TGFB1* is supported by analyses of expression in many tumors (Supplementary Fig. S17a). *MIR100HG* expression data from PanCancer Atlas were reproduced by in situ hybridization in cancer specimen (Supplementary Fig. S17b). Furthermore, clinical data correlated *MIR100HG* expression with poor survival in certain tumors and with better survival in certain others (Supplementary Fig. S18). Indeed, *MIR100HG* promotes colorectal cancer progression and predicts poor prognosis [31]. It is possible that the dual action of *MIR100HG* in cancer is linked to the dual action of TGFβ, known to have antitumor properties in certain cancers and protumorigenic actions in others, a matter worth examining in future studies.

## Materials and methods

### Reagents

Recombinant human TGFβ1, 1–5 ng/ml (PeproTech EC Ltd, London, UK) with 4 mM HCl/0.1% BSA as a vehicle, GW6604 (5 μM, Ludwig Cancer Research Ltd, New York, USA), LY2157299 (5 μM), doxorubicin (0.7 µM), taxol (0.7 µM), and 5´-fluorouracil (6.25 µM) (Sigma-Aldrich, Stockholm, Sweden), with dimethyl sulfoxide as a vehicle, were added to cells.

### Plasmids

Human *MIR100HG* (NR_024430.2) was amplified using total RNA from MCF10A cells, cDNA synthesis was synthesized via PrimeScript (Takara Bio-Europe, Saint-Germain-en-Laye, France), and PCR-amplified. The cDNA (3129 bp) was cloned in *Hind*III/*Eco*RI sites of pcDNA3 (Supplementary Fig. S4a), and sequenced (primers listed in Supplementary Table S1).

### Transfections

SiRNAs/ASOs (20–25 nM; Supplementary Table S2) were transiently transfected once or twice sequentially using siLentFect (Bio-Rad Laboratories, Solna, Sweden) targeting mRNAs or using ASOs; Dharmafect-1 (Dharmacon/VWR, Uppsala, Sweden) targeting lncRNAs; lipofectamine-3000 (ThermoFisherScientific, Stockholm, Sweden) when transfecting plasmids (pcDNA3-MIR100HG, pcDNA3), cotransfecting siRNAs and plasmids or transfecting MirVana miRNA mimics and miRNA inhibitors (Supplementary Table S3; ThermoFisherScientific), each at 10 nM. Upon transfection for 24 h, cells switched to starvation medium (0.1–1% fetal bovine serum (FBS, Biowest, Esbjerg, Denmark) in Dulbecco’s modified Eagle’s medium (DMEM, Sigma-Aldrich)) and TGFβ1 stimulation for 24 h (total period of 48 h).

### Viability assays

Cells (3000/well) seeded in 96-well plates, transfected with ASOs or plasmids, were monitored at 48 h by MTS assay, following the manufacturer’s protocol (Promega Biotech AB, Nacka, Sweden). For cytotoxicity analysis, IC_50_ curves for each drug were established in PC3U cells under transfection conditions prior to final experiments. Luminescence units from treated cells were normalized against controls. Graphs show average values of % viability with standard deviations of at least three biological experiments.

### Wound-healing assay

Cells transfected with ASOs or plasmids for 48 h were seeded (3 × 10^4^ cells/well) in the complete medium into Culture-Insert-2 (Ibidi GmbH, Gräfelfing, Germany). Confluent cell layers were starved in FBS-free medium for 16 h, the silicone insert was removed, detached cells were removed by washing twice with PBS and vehicle, or 5 ng/ml TGFβ1 were added in fresh medium. Wound closure was observed at 0 and 15 h using a Zeiss Axioplan microscope (objective ×10) with MRC digital camera. Wound surface area was quantified by ImageJ-1.47 v as a percentage of open wound per condition.

### qPCR, microarray, and database analysis

RNA extraction, reverse transcription, and qPCR were performed as described [12] with indicated primers (Supplementary Table S1). Using RT²-Profiler™ PCR Array (330231/PAHS-035Z, Qiagen, Sollentuna, Sweden), the expression of 84 human TGFβ/BMP-Signaling-Pathway genes was measured. RNA expression was calculated based on the 2^−ΔΔCt^ method, normalized to reference genes (*GAPDH*, *HPRT1*, *18S-rRNA, TBP1*) and graphed as averages with standard deviations of at least three biological experiments. Affymetrix transcriptomic data (GSE23952, GSE114761) deposited in the Gene Expression Omnibus (GEO) from NCBI were retrieved and analyzed using the GEO2R web tool. Suitability for direct comparison was assessed by sample value distribution and verification as median-centered. Adjusted *P* values were calculated via the Benjamini–Hochberg false discovery rate method.

### TaqMan assays

Small (<200 nt) RNA isolated by the NucleoSpin miRNA kit (Macherey-Nagel, Solna, Sweden) was used for TaqMan advanced miRNA assays (Supplementary Table S4) according to the manufacturer’s protocol (ThermoFisherScientific). qPCR was performed on a CFX96 cycler (Bio-Rad Laboratories). miRNA expression was calculated based on the 2^−ΔΔCt^ method, normalized to reference (*miR-191-5p*, *miR-361-5p*) miRNAs, and graphed as averages with standard deviations of at least three biological experiments.

### In situ hybridization

Formalin-fixed, paraffin-embedded tissues from melanoma, glioma, and lung adenocarcinoma patients from the Human Protein Atlas project (https://www.proteinatlas.org/) were hybridized in situ for *MIR100HG* detection using RNAscope Assays (Advanced Cell Diagnostics, Newark, CA, USA) [32].

### RNA-binding protein immunoprecipitation

RIP was performed according to the Magna-RIP^TM^ RNA-binding protein immunoprecipitation kit (Millipore/Merck, Stockholm, Sweden) as described [12]. Beads loaded with 5 μg of anti-HuR antibody (Supplementary Table S5) or normal mouse IgG (Millipore/Merck) and primers (Supplementary Table S1) were used. Graphs show average values of relative normalized levels (% input) or enrichment relative to IgG control with standard deviations of at least three biological experiments.

### Luciferase assays

Luciferase assays in HaCaT or PC3U cells transiently transfected with the CAGA_12_-luciferase promoter reporter and siRNA pools, pcDNA3-MIR100HG, or miRNA mimics were performed using the firefly and renilla dual-luciferase Assay kit (Biotium, Fremont, CA, USA) as described [12]. Relative normalized luciferase activity is expressed as averages from triplicate determinations, with standard deviations. Each experiment was repeated at least twice.

### ChIP

ChIP experiments were performed as described [12], with 3 μg of anti-Smad2/3 antibody (BD Biosciences-Europe, Stockholm, Sweden), normal mouse IgG (Millipore/Merck), and primers for qPCR of precipitated *MIR100HG* and *SERPINE1* DNAs (Supplementary Table S1).

### Immunoblotting

Protein extraction, nucleocytoplasmic fractionation followed by RNA extraction, protein quantification, and immunoblotting was performed as described [12], with primary antibodies (Supplementary Table S5) and densitometric quantification performed using ImageJ-1.47 (National Institutes of Health, MD, USA). Protein-band density normalized against the corresponding loading control (α-TUBULIN, β-ACTIN, or GAPDH) is expressed as 1 under basal or control conditions. Phosphoprotein and cleaved-protein density was normalized to the corresponding total protein.

### Thymidine-incorporation assay

HaCaT cells transiently transfected with siRNAs or miRNA mimics were seeded in 1% FBS/DMEM and treated with TGFβ1 for 24 h. Thymidine-incorporation assays were performed as described [12]. Average values with standard deviation of triplicate repeats for each condition are plotted from experiments repeated twice.

### ELISA

PC3U or A549-conditioned media were concentrated 50× through Amicon Ultra-15 centrifugal filters (Merck/Millipore) at 3000× *g* for 15 min at 4 °C or used without concentration (for certain PC3U experiments). Secreted mature TGFβ1 was measured using the human TGFβ1-Duoset ELISA kit according to the manufacturer’s instructions (R&D Systems, Oxon, UK).

### PanCancer Atlas and cholangiocarcinoma cohort analysis

The cBioPortal for Cancer Genomics [33, 34] was used to retrieve RNA-seq data from cancer patients and gene coexpression analyses of *MIR100HG* in carcinomas. All gene expressions were equally weighted. For *MIR100HG*-*TGFB1* coexpression analysis in the intrahepatic cholangiocarcinoma cohort from the French national liver biobank network, tissues were acquired as described [35], after written informed consent from all patients and study protocol approval by the local ethics committee and institutional review board of INSERM (IRB00003888). Pearson correlation value was calculated as described [35]. Survival Kaplan–Meier plots were generated using the OncoLnc platform [22].

### miRNA target analysis

Targets of *let-7a-5p* and *let-7a-2–3p* miRNAs were predicted using the DIANA microT-CDS algorithm [20]. For pathway prediction among *let-7a-2–3p* targets, DIANA mirPathv.3 was used (*p*-value threshold 0.001, microT threshold 0.8). For KEGG pathway prediction of common and unique targets of *let-7a-5p* and *let-7a-2–3p*, Enrichr (https://amp.pharm.mssm.edu/Enrichr/#) was used.

### Cell culture

Cell and media information is listed in Supplementary Table S6. Cells were free of mycoplasma (tested every 2 months) and authenticated using PCR single-locus technology (Eurofins, Uppsala, Sweden).

### Statistics

The results are shown as mean values from *n* = 3 or *n* = 2 independent biological experiments. Error bars represent standard deviations. Each biological experiment included triplicate or quintuplicate technical repeats. Comparisons were performed using a two-tailed paired Student’s *t* test and statistical significance is represented by stars (*P < 0.05, **P < 0.01, ***P < 0.001).

## Supplementary information

Supplementary information
