# Supplementary material for: The noncoding MIR100HG RNA enhances the autocrine function of transforming growth factor β signaling
Source: Oncogene. 2021 May 4;40(21):3748–65. doi: 10.1038/s41388-021-01803-8 (PMC8154591; doi:10.1038/s41388-021-01803-8)
Supplement: Supplementary file 1 — Supplementary information [file 41388_2021_1803_MOESM1_ESM.pdf]

**The non-coding *MIR100HG* RNA acts downstream of transforming growth factor β signaling and enhances its autocrine function**

Panagiotis Papoutsoglou, Dorival Mendes Rodrigues-Junior, Anita Morén, Andrew Bergman, Fredrik Pontén, Cédric Coulouarn, Laia Caja, Carl-Henrik Heldin and Aristidis Moustakas

## Supplementary Information

## Supplementary Tables

**Suppl. Table S1.** List of DNA oligonucleotides (Fw: forward; Rev: reverse)

| **Cloning primers** | **Sequence** |
|---|---|
| *MIR100HG* | Fw, AAAAAAAGCTTGGGGAGCCGCCGGAGGG CAGC<br>Rev, TTTTTGAATTCTTCACAGTTGATGCAACA TTGCTTTTATTGGGGGTTAG |
| **Sequencing primers** | **Sequence** |
| pcDNA3 CMV promoter | Fw, 5’-GTCAATGGGAGTTTGTTTTGG-3’ |
| pcDNA3 BGH polyA R1149 | Rev, 5’-GCATCGCATTGTCTGAGTAG-3’ |
| *MIR100HG* sequence 1 | 5’-GAGACTGCTATATTGATGGACTC-3’ |
| *MIR100HG* sequence 2 | 5’-GGGAAGAAAGTTATAAAACAGG-3’ |
| *MIR100HG* sequence 3 | 5’-GGGGAATGAAGTTTATCAGCATTTC-3’ |
| *MIR100HG* sequence 4 | 5’-GTGCTTCTGCACGAATGTTTATAC-3’ |
| **RT-qPCR primers** | **Sequence** |
| *MIR100HG* | Fw, AACTTGGCTTCCTCGCTTCT<br>Rev, CTTTGTCCTTGCACTGGGGA |
| *SERPINE1* | Fw, GAGACAGGCAGCTCGGATTC<br>Rev, GGCCTCCCAAAGTGCATTAC |
| *FN1* | Fw, CCCAGACTTATGGTGGCAATTC<br>Rev, AATTTCCGCCTCGAGTCTGA |
| *SMAD7* | Fw, ACCCGATGGATTTTCTCAAACC<br>Rev, GCCAGATAATTCGTTCCCCCT |
| *CDH2* | Fw, CCTGCTTCAGGCGTCTGTAGA<br>Rev, TCATGCACATCCTTCGATAAGACT |
| *HPRT1* | Fw, CCCTGGCGTCGTGATTAGT<br>Rev, CACCCTTTCCAAATCCTCAGC |
| *HuR* | Fw, GCCGTCACCAATGTGAAAGT<br>Rev, CCATCGCGGCTTCTTCATAG |
| *GAPDH* | Fw, GGAGTCAACGGATTTGGTCGTA<br>Rev, GGCAACAATATCCACTTTACCA |
| *18S rRNA* | Fw, GTAACCCGTTGAACCCCATT |

| | |
|---|---|
| | Rev, CCATCCAATCGGTAGTAGCG |
| *TBP* | Fw, GAGCTGTGATGTGAAGTTTCC<br>Rev, TCTGGGTTTGATCATTCTGTAG |
| *TGFB2-AS1* | Fw, AGGGAGTGTGGAAATGAGG<br>Rev, GGGTTTGGGAGTACATTCAAC |
| *SNAI1* | Fw, CACTATGCCGCGCTCTTTC<br>Rev, GCTGGAAGGTAAACTCTGGATTAGA |
| *CDKN1A* | Fw, CTGCCCAAGCTCTACCTTCC<br>Rev, CAGGTCCACATGGTCTTCCT |
| *CDKN2B* | Fw, TGGACCTGGTGGCTACGAAT<br>Rev, AGGGCCTAAGTTGTGGGTTCA |
| *JUN* | Fw, GCTAACGCAGCAGTTGCAAAC<br>Rev, CCGTCGCAACTTGTCAAGTTC |
| *BMPR2* | Fw, ATGCAGCCATAAGCGAGGTT<br>Rev, CCCCTGGGAAGAGGTCTGTA |
| *SOX4* | Fw, CGCGTGATGAAGACAGAAGGCTCCG<br>Rev, AAACGGGAATTCGCCTGCGTGG |
| *THBS1* | Fw, TTGTCTTTGGAACCACACCA<br>Rev, CTGGACAGCTCATCACAGGA |
| *TGFBI* | Fw, AATCGCTTTAGCATGCTGGT<br>Rev, CAAGAGTCTGCTCCGTTCTC |
| *GDF2* | Fw, AGAACGTGAAGGTGGATTTCC<br>Rev, CGCACAATGTTGGACGCTG |
| *ID1* | Fw, GGACGAGCAGCAGGTAAACG<br>Rev, TGCTCACCTTGCGGTTCTG |
| *TGFB1* | Fw, GCCTTTCCTGCTTCTCATGG<br>Rev, TCCTTGCGGAAGTCAATGTAC |
| **ChIP genomic DNA primers** | **Sequence** |
| *MIR100HG promoter* | Fw, AGCAAACACATTTCAGGCAGT<br>Rev, GGCTACCTGACTGATGAGTG |
| *SERPINE1 promoter* | Fw, GCAGGACATCCGGGAGAGA<br>Rev, CCAATAGCCTTGGCCTGAGA |

**Suppl. Table S2.** List of siRNAs and ASOs

| RNAi reagent | Product ID |
|---|---|
| siSMAD2 | ON-TARGETplus Human SMAD2 siRNA SMARTpool, L-003561-00, Dharmacon/VWR, Uppsala, Sweden |
| siSMAD3 | ON-TARGETplus Human SMAD3 siRNA SMARTpool, L-020067-00, Dharmacon/VWR, Uppsala, Sweden |
| siSMAD4 | ON-TARGETplus Human SMAD4 siRNA SMARTpool, L-003902-00, Dharmacon/VWR, Uppsala, Sweden |
| siTGFBR2 | ON-TARGETplus Human TGFBR2 siRNA SMARTpool, L-003930-00, Dharmacon/VWR, Uppsala, Sweden |
| siMIR100HG | Lincode Human MIR100HG siRNA SMARTpool, R-029813-01, Dharmacon/VWR, Uppsala, Sweden |
| siMIR100HG#9 | Lincode Human MIR100HG siRNA Individual, N-029813-09, Dharmacon/VWR, Uppsala, Sweden |
| siTGFβ1 | Genesolution Hs_TGFB1_6 siRNA, Cat. No. S102662912, Qiagen, Sollentuna, Sweden |
| siHuR/ELAVL1#1 | ON-TARGETplus Human ELAVL1 siRNA, J-003773-08, Dharmacon/VWR, Uppsala, Sweden |
| siHuR/ELAVL1#2 | ON-TARGETplus Human ELAVL1 siRNA, J-003773-09, Dharmacon/VWR, Uppsala, Sweden |
| siHuR/ELAVL1#3 | ON-TARGETplus Human ELAVL1 siRNA, J-003773-10, Dharmacon/VWR, Uppsala, Sweden |
| siHuR/ELAVL1#4 | ON-TARGETplus Human ELAVL1 siRNA, J-003773-11, Dharmacon/VWR, Uppsala, Sweden |
| Antisense LNA™-GapmeR (ASO) targeting *MIR100HG*/ NR_024430.2_1 | 5´-ATCGATTGGTTAGTGT-3´, nr.-339511, LG00215440-DDA, Exiqon/Qiagen, Sollentuna, Sweden |
| Control ASO | 5´-AACACGTCTATACGC-3´, nr.-33915, LG00000002-DDA, Exiqon/Qiagen, Sollentuna, Sweden |

**Suppl. Table S3.** List of miRNA-mimics and -inhibitors

| **mirVanamiRNA-mimic** | **Product ID** |
|---|---|
| *hsa-let-7a-2-3p* | MC11174 |
| *hsa-miR-125b-1-3p* | MC12582 |
| *hsa-miR-125b-5p* | MC10148 |
| mirVanamiRNA-mimic negative control#1 | 4464058 |
| **mirVanamiRNA-inhibitor** | **Product ID** |
| *hsa-let-7a-2-3p* | MH11174 |
| mirVanamiRNA-inhibitor negative control#1 | 4464076 |

**Suppl. Table S4.** List of TaqMan Advanced microRNA assays

| **TaqMan Advanced microRNA assay** | **Assay ID** |
|---|---|
| *hsa-let-7a-2-3p* | 479269_mir |
| *hsa-let-7a-5p* | 478575_mir |
| *hsa-miR-125b-1-3p* | 478665_mir |
| *hsa-miR-125b-5p* | 477885_mir |
| *hsa-miR-100-5p* | 478224_mir |
| *hsa-miR-100-3p* | 478619_mir |
| *hsa-miR-191-5p* | 477952_mir |
| *hsa-miR-361-5p* | 478056_mir |
| *MIR100 (pre-miR-100)* | *Hs04231438_s1* |
| *MIR125B1 (pre-miR-125b-1)* | *Hs04231493_s1* |
| *MIRLET7A2 (pre-miR-let-7a-2)* | *Hs04231409_s1* |

**Suppl. Table S5.** List of antibodies

| Antibody | Dilution | Source | Catalogue number |
|---|---|---|---|
| β-Actin (AC-15) | 1:1 000 | Santa-Cruz Biotechonology, Dallas, TX, USA | sc-69879 |
| Caspase 3 | 1:500 | Cell Signaling Technology | Ab13847 |
| Fibronectin | 1:20 000 | Sigma-Aldrich, Stockholm, Sweden | F3648 |
| GAPDH | 1:20 000 | Ambion | AM4300 |
| HuR (3A2) | 1:500 | ThermoFisher/Invitrogen | 39-0600 |
| Lamin B1 | 1:1 000 | Abcam, Cambridge, UK | Ab16048 |
| N-Cadherin | 1:1 000 | BD-Biosciences, Stockholm, Sweden | 610920 |
| p38 | 1:1 000 | Cell Signaling Technology, Danvers, MA, USA | 9212 |
| p-p38 | 1:1 000 | Cell Signaling Technology, Danvers, MA, USA | 9211 |
| PAI-1 | 1:1 000 | BD-Biosciences, Stockholm, Sweden | 612025 |
| PARP-1 | 1:2 000 | BD-Biosciences, Stockholm, Sweden | 51-8114KC |
| SMAD2/3 | 1:1 000 | BD-Biosciences, Stockholm, Sweden | 610843 |
| p-SMAD2 | 1:2 000 | home-made, Ludwig Cancer Research-Uppsala Branch, Sweden | - |
| SMAD3 | 1:1 000 | Cell Signaling Technology, Danvers, MA, USA | 9523S |
| p-SMAD3 | 1:1 000 | Cell Signaling Technology, Danvers, MA, USA | 9520S |
| SMAD4 | 1:1 000 | Epitomics, Inc./Abcam, Cambridge, UK | 1735-1 |
| α-Tubulin | 1:1 000 | Santa-Cruz Biotechonology, Santa Cruz, CA, USA | sc-8035 |

**Suppl. Table S6.** List of cell lines and culture media

| Cell line | Source | Medium | Medium source |
|---|---|---|---|
| A549 (CRM-CCL-185) | American Type Culture Collection (ATCC), Manassas, Virginia, USA | 10% FBS/DMEM/ 100 U/ml penicillin, 100 µg/ml streptomycin | Sigma-Aldrich, Stockholm, Sweden |
| HuCCT1 (RCB-1960) | RIKEN BioResource Center (Tsukuba-shi, Japan) | 10%FBS/RPMI-1640/ 2 mM L-glutamine/ 100 U/ml penicillin, 100 µg/ml streptomycin | Sigma-Aldrich, Stockholm, Sweden |
| PC3U | Ludwig Cancer Research Ltd, Uppsala Branch | 10%FBS/RPMI-1640/ 2 mM L-glutamine/ 100 U/ml penicillin, 100 µg/ml streptomycin | Sigma-Aldrich, Stockholm, Sweden |
| MCF10A | F.R. Miller, Fred Hutschinson Cancer Center, Seattle, USA | 5% FBS/DMEM/F12/ 100 U/ml penicillin, 100 µg/ml streptomycin | Sigma-Aldrich, Stockholm, Sweden |
| HaCaT keratinocytes | Ludwig Cancer Research Ltd, Uppsala Branch | 10% FBS/DMEM/ 100 U/ml penicillin, 100 µg/ml streptomycin | Sigma-Aldrich, Stockholm, Sweden |

## Supplementary Figures

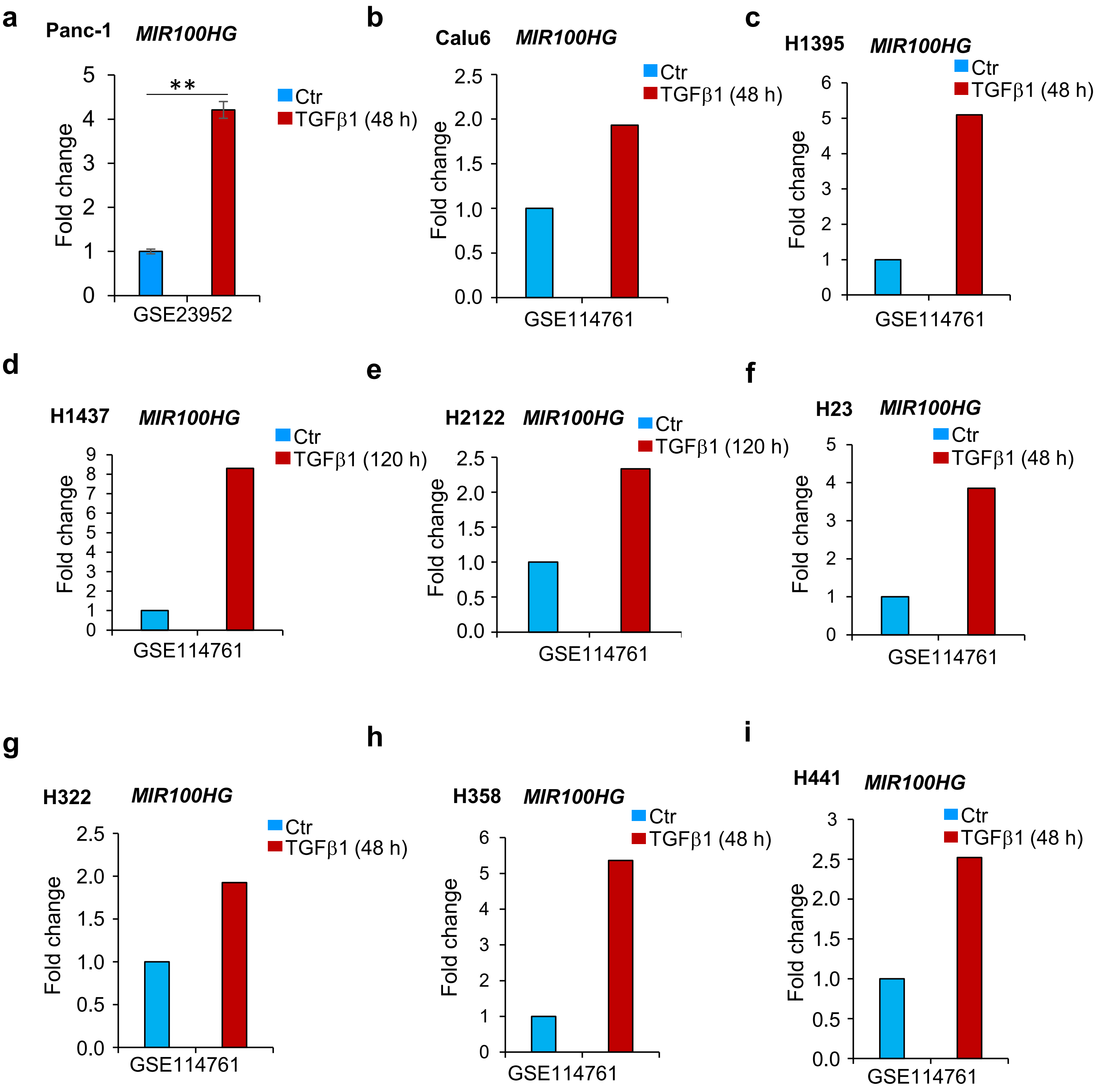
a
Panc-1 MIR100HG
Fold change
0
1
2
3
4
5
**
Ctr
TGFβ1 (48 h)
GSE23952
b
Calu6 MIR100HG
Fold change
0.0
0.5
1.0
1.5
2.0
2.5
Ctr
TGFβ1 (48 h)
GSE114761
c
H1395 MIR100HG
Fold change
0
1
2
3
4
5
6
Ctr
TGFβ1 (48 h)
GSE114761
d
H1437 MIR100HG
Fold change
0
1
2
3
4
5
6
7
8
9
Ctr
TGFβ1 (120 h)
GSE114761
e
H2122 MIR100HG
Fold change
0.0
0.5
1.0
1.5
2.0
2.5
Ctr
TGFβ1 (120 h)
GSE114761
f
H23 MIR100HG
Fold change
0
1
2
3
4
5
Ctr
TGFβ1 (48 h)
GSE114761
g
H322 MIR100HG
Fold change
0.0
0.5
1.0
1.5
2.0
2.5
Ctr
TGFβ1 (48 h)
GSE114761
h
H358 MIR100HG
Fold change
0
1
2
3
4
5
6
Ctr
TGFβ1 (48 h)
GSE114761
i
H441 MIR100HG
Fold change
0.0
0.5
1.0
1.5
2.0
2.5
3
Ctr
TGFβ1 (48 h)
GSE114761

Figure S1

**Suppl. Fig. S1.** *MIR100HG* is induced by TGFβ. **a-i** Transcriptomic data from microarray analysis for determination of *MIR100HG* expression in human Panc-1 pancreatic adenoracinoma (a) and lung adenocarcinoma (b-i) cells, in response to TGFβ1 treatment for the indicated time periods. Gene expression data are extracted from GSE23952 (a) and GSE114761 (b-i) public datasets. Error bars represent standard deviation from three biological experiments **$p$<0.01).

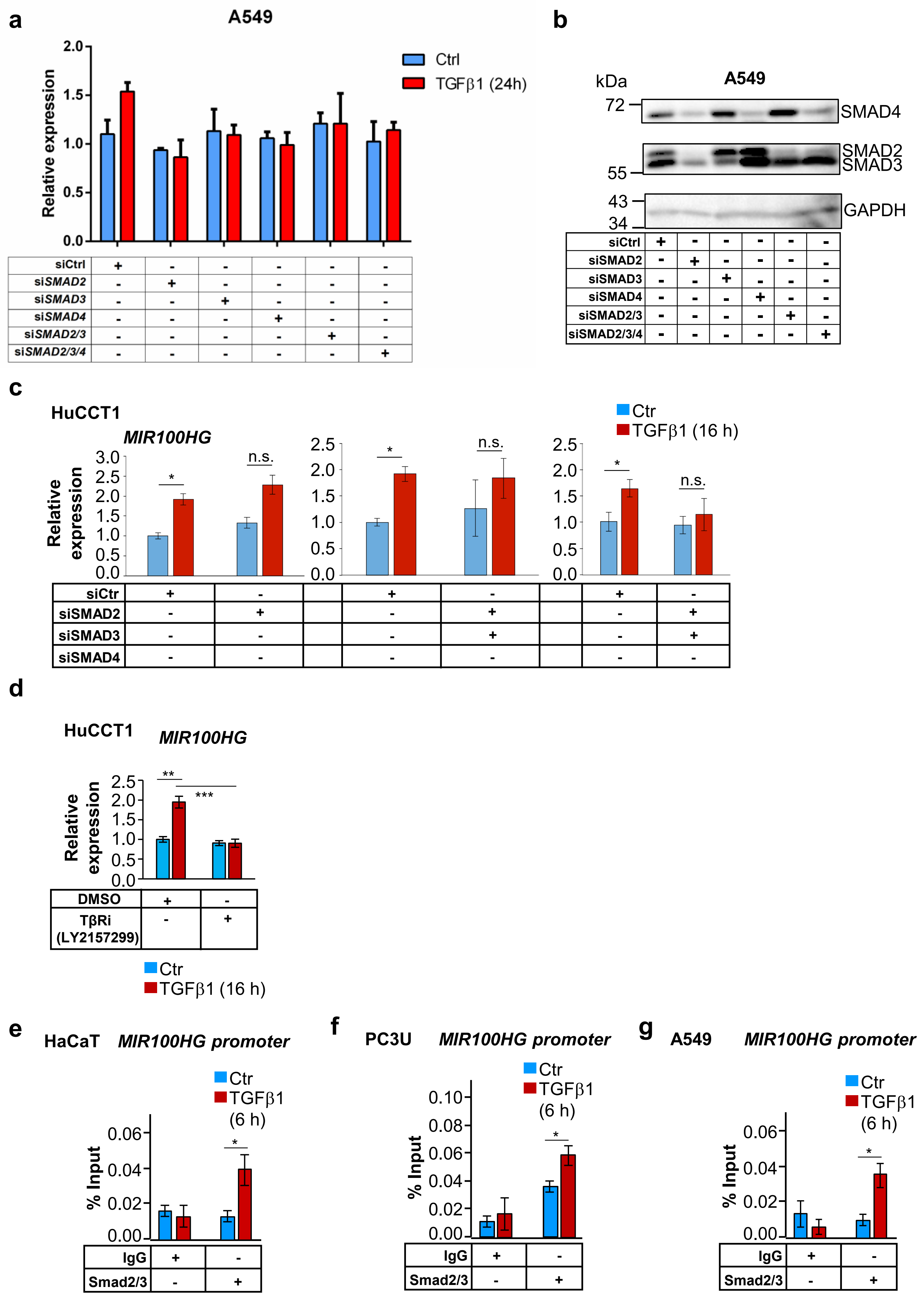

a
A549
Ctrl
TGFβ1 (24h)
Relative expression
2.0
1.5
1.0
0.5
0.0
siCtrl + - - - - -
siSMAD2 - + - - - -
siSMAD3 - - + - - -
siSMAD4 - - - + - -
siSMAD2/3 - - - - + -
siSMAD2/3/4 - - - - - +
b
kDa
A549
72
55
43
34
SMAD4
SMAD2
SMAD3
GAPDH
siCtrl + - - - - -
siSMAD2 - + - - - -
siSMAD3 - - + - - -
siSMAD4 - - - + - -
siSMAD2/3 - - - - + -
siSMAD2/3/4 - - - - - +
c
HuCCT1
MIR100HG
Ctr
TGFβ1 (16 h)
Relative expression
*
n.s.
siCtr + - + - + -
siSMAD2 - + - + - +
siSMAD3 - - - + - +
siSMAD4 - - - - - -
d
HuCCT1
MIR100HG
Relative expression
**
***
DMSO + -
TβRi (LY2157299) - +
Ctr
TGFβ1 (16 h)
e
HaCaT
MIR100HG promoter
Ctr
TGFβ1 (6 h)
% Input
*
IgG + -
Smad2/3 - +
f
PC3U
MIR100HG promoter
Ctr
TGFβ1 (6 h)
% Input
*
IgG + -
Smad2/3 - +
g
A549
MIR100HG promoter
Ctr
TGFβ1 (6 h)
% Input
*
IgG + -
Smad2/3 - +


Figure S2

**Suppl. Fig. S2.** *MIR100HG* is induced by TGFβRI-SMAD signaling. **a, c** Real time RT-qPCR for determination of *MIR100HG* in A549 (a) or HuCCT1 (c) cells transiently transfected with siRNAs targeting SMAD2, SMAD3, SMAD4 or combinations and treated or not with TGFβ1 for 24 h. Gene expression is normalized relative to the housekeeping gene *HPRT1*. Error bars represent standard deviation from three different experiments (*$p$<0.05). **b** Immunoblots corresponding to the experiment of panel (a) indicating efficiency of SMAD protein silencing in specific combinations that cover all three SMADs examined. GAPDH was used as a loading control and molecular mass (kDa) markers are indicated. **d** Real time RT-qPCR for *MIR100HG* in HuCCT1 cells treated with TβRi (LY2157299) in combination with TGFβ1 stimulation for 24 h. Gene expression is normalized relative to the housekeeping gene *TBP*. Error bars represent standard deviation from three different experiments (**$p$<0.01, ***$p$<0.001). **e-g** ChIP-qPCR analysis for SMAD2/3 occupancy to the *MIR100HG* promoter in HaCaT (e), PC3U (f) and A549 (g) cells stimulated with TGFβ or not for 6 h. Control IgG immunoprecipitation data are also shown. Error bars represent standard deviation from three different experiments (*$p$<0.05).

Figure S3

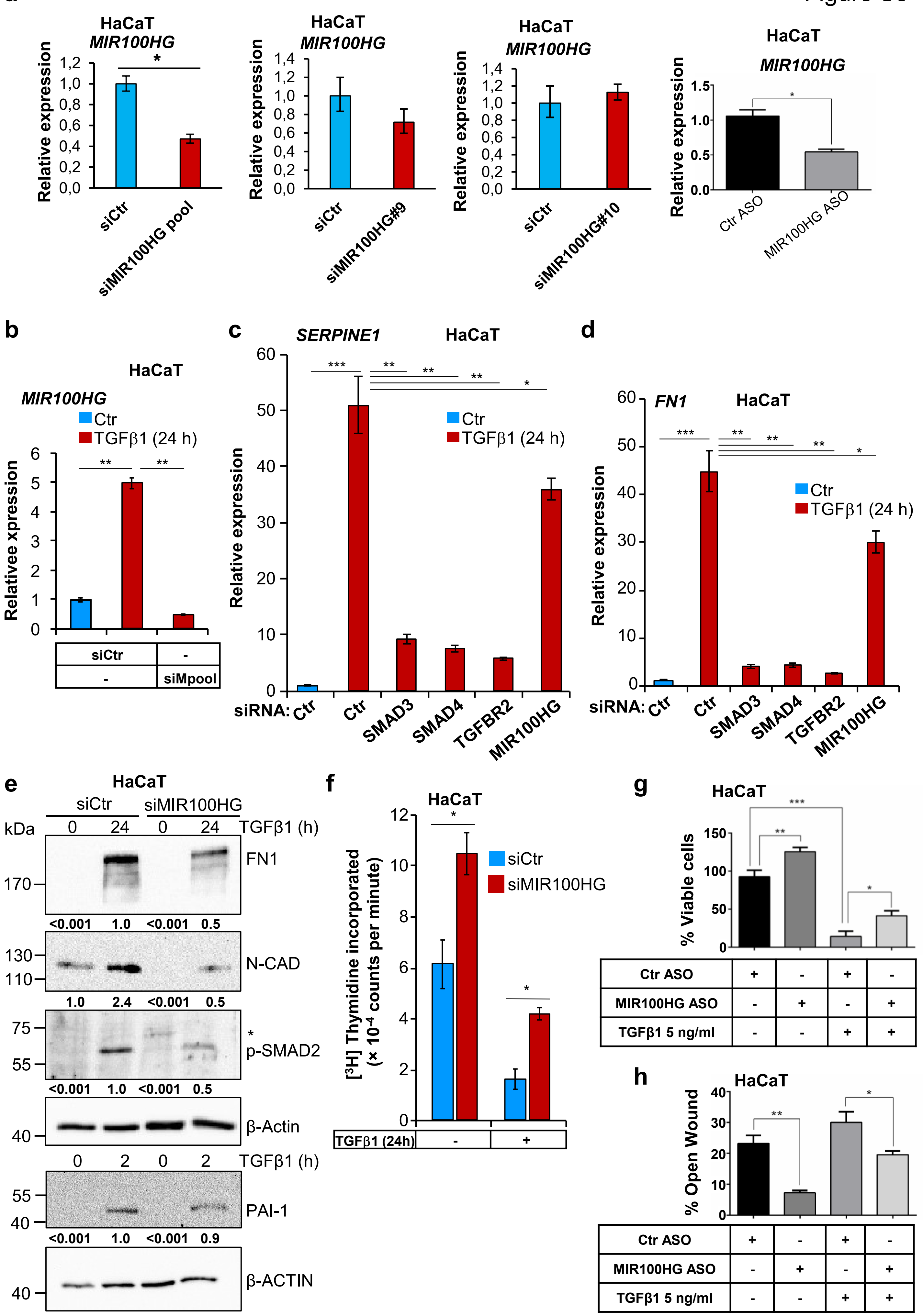

a
HaCaT
MIR100HG
Relative expression
siCtr
siMIR100HG pool
siMIR100HG#9
siMIR100HG#10
Ctr ASO
MIR100HG ASO
b
HaCaT
MIR100HG
Ctr
TGFβ1 (24 h)
Relativee xpression
siCtr
siMpool
c
SERPINE1
HaCaT
Relative expression
siRNA: Ctr
Ctr
SMAD3
SMAD4
TGFBR2
MIR100HG
d
FN1
HaCaT
e
HaCaT
siCtr
siMIR100HG
kDa
TGFβ1 (h)
FN1
N-CAD
p-SMAD2
β-Actin
PAI-1
β-ACTIN
<0.001 1.0 <0.001 0.5
1.0 2.4 <0.001 0.5
<0.001 1.0 <0.001 0.5
<0.001 1.0 <0.001 0.9
f
HaCaT
[3H] Thymidine incorporated (× 10-4 counts per minute)
siCtr
siMIR100HG
TGFβ1 (24h)
g
HaCaT
% Viable cells
Ctr ASO
MIR100HG ASO
TGFβ1 5 ng/ml
h
HaCaT
% Open Wound

**Suppl. Figure S3.** Silencing *MIR100HG* decreases TGFβ-signaling responses in HaCaT cells. **a** Real time RT-qPCR for detection of *MIR100HG* expression in HaCaT cells transiently transfected with the indicated siRNApool-of-4, specific siRNAs#9 or #10 or anti-MIR100HG ASO. Gene expression is normalized relative to the housekeeping gene *HPRT1*. Error bars represent standard deviation from three different experiments (**p*<0.05). **b** Real time RT-qPCR for detection of *MIR100HG* expression in HaCaT cells transiently transfected with the control of *MIR100HG*-specific siRNApool-of-4 and stimulated with TGFβ1 or not for 24 h. Gene expression is normalized relative to the housekeeping gene *18S rRNA*. Error bars represent standard deviation from three different experiments (***p*<0.01). **c**, **d** Real time RT-qPCR for detection of *SERPINE1* (c) and *FN1* (d) expression in HaCaT cells transiently transfected with the indicated siRNA pools and stimulated with TGFβ1 or not for 24 h. Gene expression is normalized relative to the housekeeping gene *HPRT1*. Error bars represent standard deviation from three different experiments (**p*<0.05, ***p*<0.01, ****p*<0.001). **e** Representative immunoblots out of three independent experiments for expression of FN1, N-CAD and phosphorylated SMAD2 (p-SMAD2) (top immunoblot) and of PAI-1 in HaCaT cells transiently transfected with Control siRNA (siC) or siMIR100HG#9 and treated with TGFβ1 for 24 h (top) or 2 h (bottom). β-ACTIN was used as a loading control and molecular mass (kDa) markers are indicated along with densitometric values of normalized band intensity. A star indicates a non-specific protein band recognized by the antibody. **f** $^{3}$H-thymidine incorporation assay in HaCaT cells transiently transfected with control siRNA (siCtr) or siMIR100HGpool-of-4 and treated or not with TGFβ1 for 24 h. Error bars represent standard deviation from three different experiments (**p*<0.05, ***p*<0.01). **g** Cell viability/proliferation assay with HaCaT cells transiently transfected with negative control (Ctr) or anti-MIR100HG ASO

(as shown in panel a) and treated with TGFβ1 for 24 h. Error bars represent standard deviation from three independent experiments (**p*<0.05, ***p*<0.01, ****p*<0.001). **h** Cell migration assay with HaCaT cells transiently transfected with negative control (Ctr) or anti-MIR100HG ASO (as shown in panel a) and treated with TGFβ1 for 24 h. Error bars represent standard deviation from three independent experiments (**p*<0.05, ***p*<0.01).

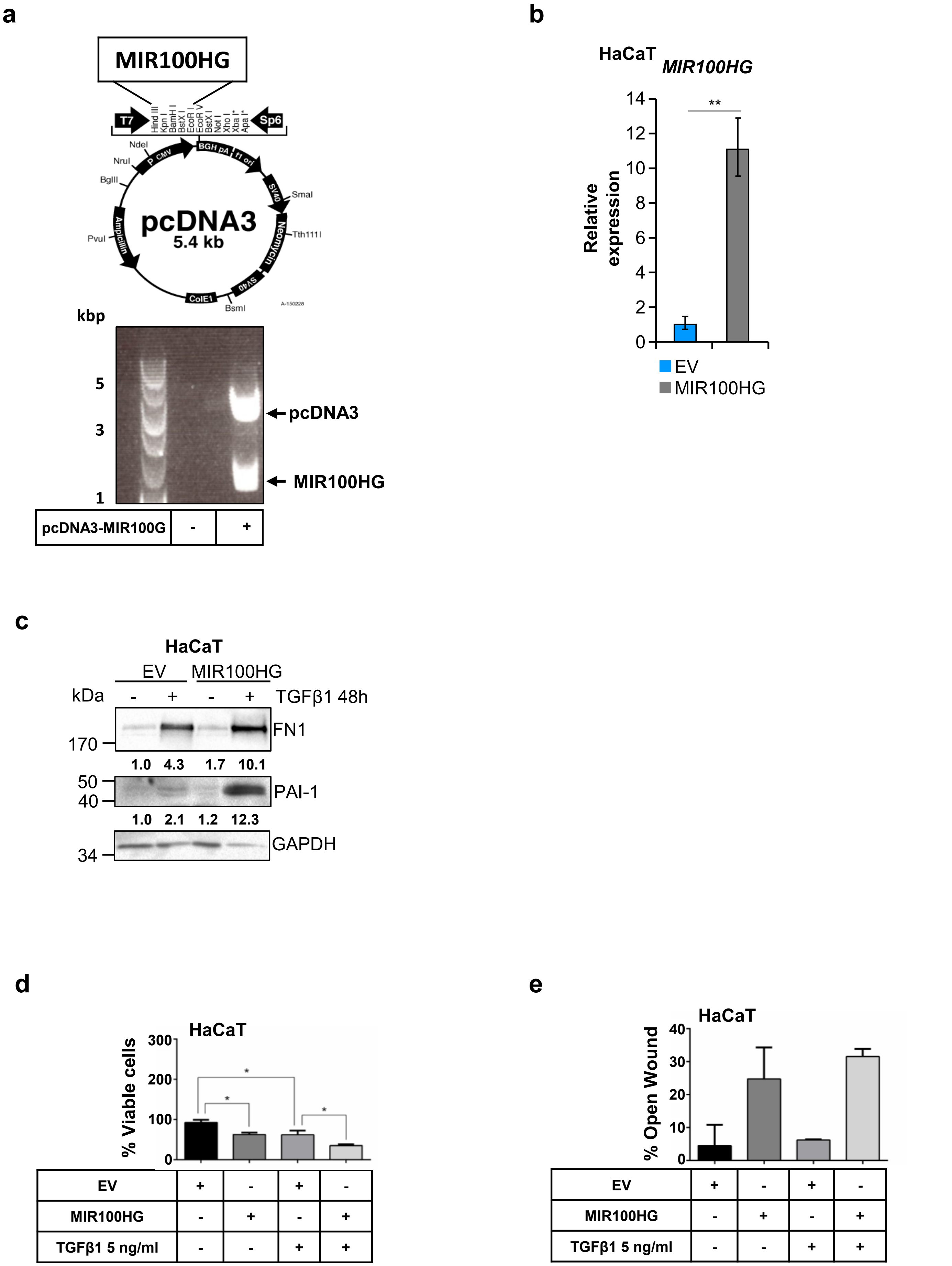
a
MIR100HG
T7
Sp6
Hind III
Kpn I
BamH I
BstX I
EcoR I
EcoR V
BstX I
Not I
Xho I
Xba I*
Apa I*
NdeI
NruI
BglII
PvuI
P CMV
BGH pA
f1 ori
SV40
SmaI
Neomycin
Tth111I
SV40
BsmI
ColE1
Ampicillin
pcDNA3
5.4 kb
kbp
5
3
1
pcDNA3
MIR100HG
pcDNA3-MIR100G
-
+
b
HaCaT
MIR100HG
Relative expression
**
EV
MIR100HG
c
HaCaT
EV
MIR100HG
kDa
TGFβ1 48h
FN1
170
1.0 4.3 1.7 10.1
PAI-1
50
40
1.0 2.1 1.2 12.3
GAPDH
34
d
HaCaT
% Viable cells
EV + - + -
MIR100HG - + - +
TGFβ1 5 ng/ml - - + +
e
HaCaT
% Open Wound
EV + - + -
MIR100HG - + - +
TGFβ1 5 ng/ml - - + +

Figure S4

**Suppl. Figure S4.** Construction and efficiency of the pcDNA3-MIR100HG expression vector. **a** Schematic representation of the recombinant pcDNA3-MIR100HG expression vector (top). Efficient ligation of the *MIR100HG* insert cDNA to pcDNA3 plasmid vector was verified by double digestion with restriction enzymes, followed by agarose gel electrophoresis (bottom). Molecular size (in kbp) marker ladder is shown in the first lane. **b** Real time RT-qPCR for *MIR100HG* expression in HaCaT cells transiently transfected with pcDNA3-MIR100HG expressing vector. Gene expression is normalized relative to the housekeeping gene *18S rRNA*. Error bars represent standard deviation from three different experiments (**$p$<0.01). **c** Representative immunoblot out of three independent experiments for expression of FN1 and PAI-1 in HaCaT cells transiently transfected with empty vector (EV) or pcDNA3-MIR100HG (as shown in panel b) and treated with TGFβ1 for 48 h. GAPDH was used as a loading control and molecular mass (kDa) markers are indicated along with densitometric values of normalized band intensity. **d** Cell viability/proliferation assay with HaCaT cells transiently transfected with empty vector (EV) or pcDNA3-MIR100HG (as shown in panel b) and treated with TGFβ1 for 24 h. Error bars represent standard deviation from three independent experiments (*$p$<0.05). **e** Cell migration assay with HaCaT cells transiently transfected with empty vector (EV) or pcDNA3-MIR100HG (as shown in panel b) and treated with TGFβ1 for 24 h. Lack of stars indicates lack of statistical significance based on three independent experiments.

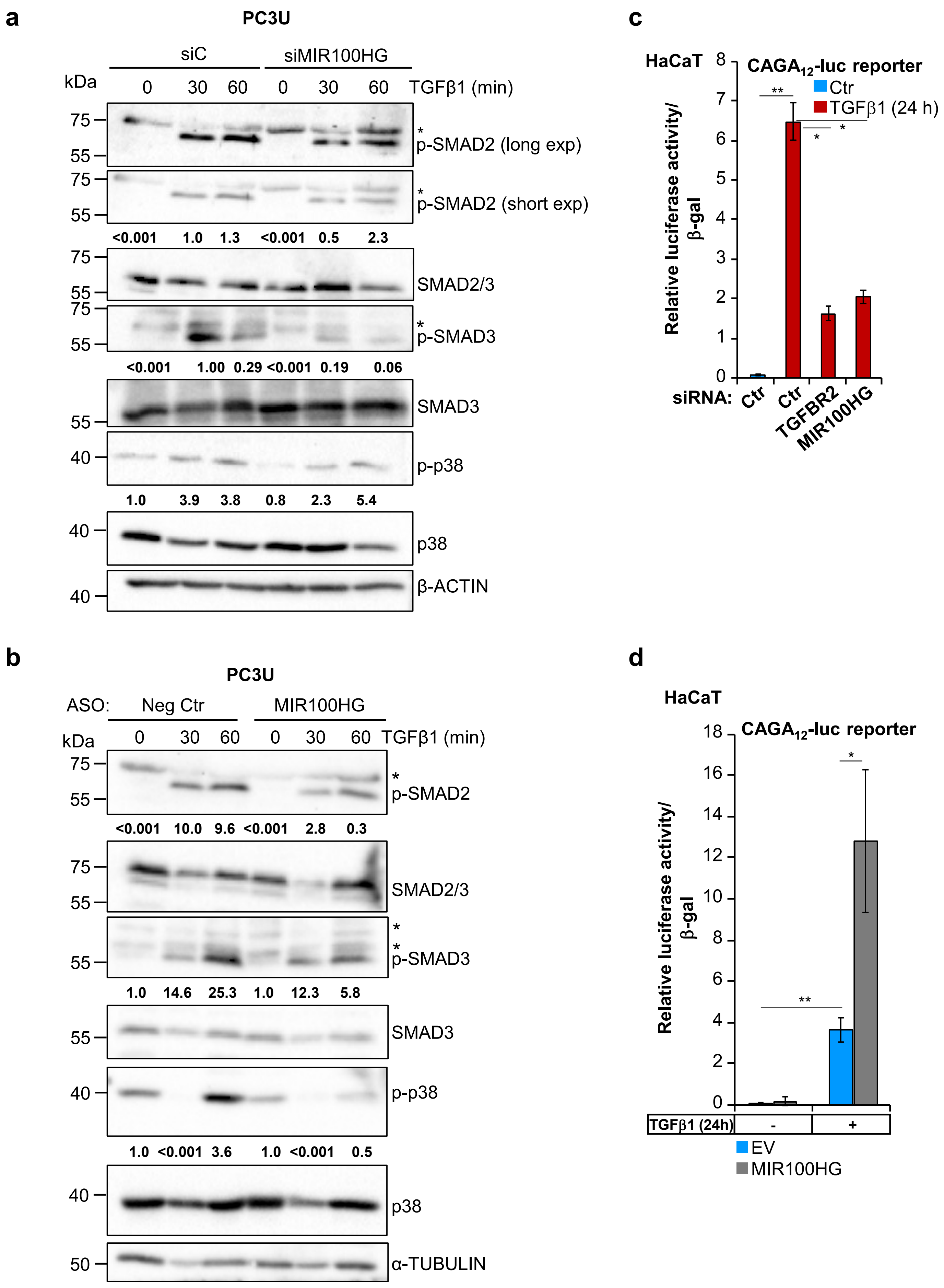
a
PC3U
siC
siMIR100HG
kDa
0 30 60 0 30 60 TGFβ1 (min)
75
55
p-SMAD2 (long exp)
p-SMAD2 (short exp)
<0.001 1.0 1.3 <0.001 0.5 2.3
SMAD2/3
p-SMAD3
<0.001 1.00 0.29 <0.001 0.19 0.06
SMAD3
40
p-p38
1.0 3.9 3.8 0.8 2.3 5.4
p38
β-ACTIN
b
PC3U
ASO: Neg Ctr MIR100HG
kDa
0 30 60 0 30 60 TGFβ1 (min)
p-SMAD2
<0.001 10.0 9.6 <0.001 2.8 0.3
SMAD2/3
p-SMAD3
1.0 14.6 25.3 1.0 12.3 5.8
SMAD3
p-p38
1.0 <0.001 3.6 1.0 <0.001 0.5
p38
50
α-TUBULIN
c
HaCaT
CAGA12-luc reporter
Ctr
TGFβ1 (24 h)
Relative luciferase activity/ β-gal
siRNA: Ctr Ctr TGFBR2 MIR100HG
d
HaCaT
CAGA12-luc reporter
Relative luciferase activity/ β-gal
TGFβ1 (24h) - +
EV
MIR100HG

Figure S5

**Suppl. Fig. S5.** *MIR100HG* regulates basic TGFβ receptor signaling. **a, b** Representative immunoblots, out of three independent experiments, for protein expression levels of phosphorylated SMAD2 (p-SMAD2), SMAD2/3, phosphorylated SMAD3 (p-SMAD3), SMAD3, phosphorylated p38 (p-p38) and p38 in PC3U cells transiently transfected with Control siRNA (siC) or siMIR100HG#9 (a) and with negative control or anti-MIR100HG ASO (b) (as shown in Fig. 3a, f) and treated with TGFβ1 for the indicated time periods. β-ACTIN was used as a loading control and molecular mass (kDa) markers are indicated along with densitometric values of normalized band intensity. Two different exposures of the p-SMAD2 immunoblot are shown (a). Stars indicate non-specific protein bands recognized by the antibody. **c** silencing of *TGFBR2* (positive control) and *MIR100HG* by siRNApools-of-4, followed by monitoring TGFβ signaling output after 24 h, using a $CAGA_{12}$-luciferase reporter assay in HaCaT cells. Error bars represent standard deviation from three different experiments (*$p$<0.05, **$p$<0.01). **d** $CAGA_{12}$-luciferase assay in HaCaT cells transiently overexpressing *MIR100HG* (as shown in Suppl. Fig. S4b) and in the presence or absence of TGFβ1 stimulation for 24 h. Error bars represent standard deviation from three different experiments (*$p$<0.05, **$p$<0.01).

| TGFβ/BMP pathway Array<br><br>Basal Gene Expression ($C_t$ value) | | | | | | | | | | | |
|---|---|---|---|---|---|---|---|---|---|---|---|
| **ACVR1** (21.52) | **ACVR2A** (21.50) | **ACVRL1** (31.14) | **AMH** (31.75) | **AMHR2** (27.41) | **ATF4** (16.94) | **BAMBI** (22.37) | **BGLAP** (23.64) | **BMP1** (23.86) | **BMP2** (27.72) | **BMP3** (23.10) | **BMP4** (25.37) |
| **BMP5** (27.04) | **BMP6** (27.38) | **BMP7** (33.62) | **BMPER** (28.76) | **BMPR1A** (22.32) | **BMPR1B** (22.86) | **BMPR2** (21.18) | **CDKN1A** (21.00) | **CDKN1B** (22.44) | **CDKN2B** (20.30) | **CHRD** (29.61) | **COL1A1** (25.60) |
| **COL1A2** (29.27) | **DCN** (24.60) | **DLX2** (22.76) | **EMP1** (20.82) | **ENG** (25.52) | **FOS** (25.86) | **FST** (18.55) | **GADD45B** (20.84) | **GDF2** (25.17) | **GDF3** (32.78) | **GDF5** (29.14) | **GDF6** (30.15) |
| **GDF7** (32.31) | **GSC** (31.59) | **HERPUD1** (18.80) | **HIPK2** (21.43) | **ID1** (20.87) | **ID2** (20.79) | **IFRD1** (20.10) | **IGF1** (28.73) | **IGFBP3** (21.76) | **IL6** (21.07) | **INHA** (27.40) | **INHBA** **(18.15)** |
| **INHBB** (24.08) | **JUN** (21.36) | **JUNB** (22.11) | **LEFTY1** (26.14) | **LTBP1** (17.32) | **LTBP2** (24.60) | **MECOM** (22.90) | **MYC** (18.93) | **NODAL** (29.08) | **NOG** (25.59) | **PDGFB** (25.63) | **PLAU** (18.73) |
| **RUNX1** (20.98) | **SERPINE1** (19.28) | **SMAD1** (20.24) | **SMAD2** (20.32) | **SMAD3** (21.65) | **SMAD4** (21.13) | **SMAD5** (19.81) | **SMAD7** (26.31) | **SMURF1** (21.88) | **SOX4** (25.61) | **STAT1** (19.13) | **TGFB1** (19.62) |
| **TGFB1I1** (23.31) | **TGFB2** (21.10) | **TGFB3** (24.20) | **TGFBI** (27.60) | **TGFBR1** (22.83) | **TGFBR2** (20.62) | **TGFBR3** (24.78) | **TGFBRAP1** (21.72) | **TGIF1** (20.05) | **THBS1** (18.96) | **TNFSF10** (25.76) | **TSC22D1** (19.27) |
| **ACTB** (12.12) | **B2M** (15.10) | **GAPDH** (14.69) | **HPRT1** (20.31) | **RPLP0** (13.65) | **HGDC** (30.04) | **RTC** (29.85) | **RTC** (29.52) | **RTC** (29.43) | **PPC** (17.92) | **PPC** (18.04) | **PPC** (17.77) |

Figure S6

**Suppl. Figure S6.** RT2 profiler PCR array of the human TGFβ/BMP signaling pathway and TGFβ1 quantification. Diagram of the 96-well plate with specific probes for 84 genes classified as “TGFβ/BMP signaling” based on bioinformatic annotation of signaling pathways (84 gene probe sets), 5 control “house-keeping” genes (ACTB (β-ACTIN), B2M (β2-MACROGLOBULIN), GAPDH, HPRT1 and RPLP0 (RIBOSOMAL PROTEIN LATERAL STALK SUBUNIT P0); 1 control genomic DNA gene (HGDC (R)-HYDROXYGLUTARYL-CoA DEHYDRATASE ACTIVATOR)), 3 reverse transcriptase control probes (RTC) and 3 positive PCR controls (PPC). Each well indicates the probe name and the associated $C_t$ value derived from one representative assay.

**a**

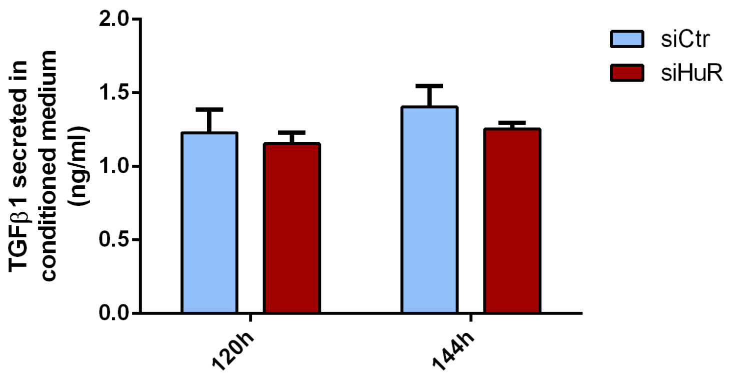
PC3U
TGFβ1 secreted in conditioned medium (ng/ml)
2.0
1.5
1.0
0.5
0.0
120h
144h
siCtr
siHuR

**b**

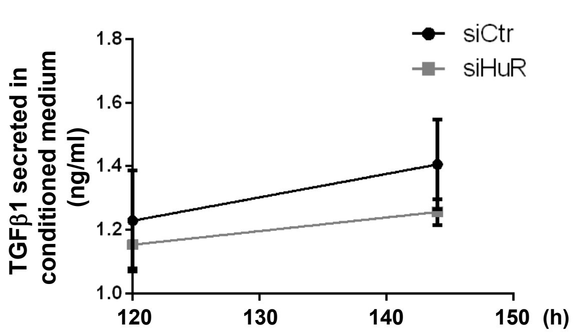
PC3U
TGFβ1 secreted in conditioned medium (ng/ml)
1.8
1.6
1.4
1.2
1.0
120
130
140
150
(h)
siCtr
siHuR

Figure S7

**Suppl. Figure S7.** Silencing HuR alone does not significantly reduce TGFβ1 secretion. **a, b** Secreted mature TGFβ1 ligand quantification in the conditioned medium of PC3U cells transiently transfected with negative control or *HuR*-specific siRNA (as shown in Fig. 7c) for the indicated time periods and in the absence of TGFβ stimulation. Error bars represent standard deviation from three different experiments. Absence of stars indicate absence of significant differences.

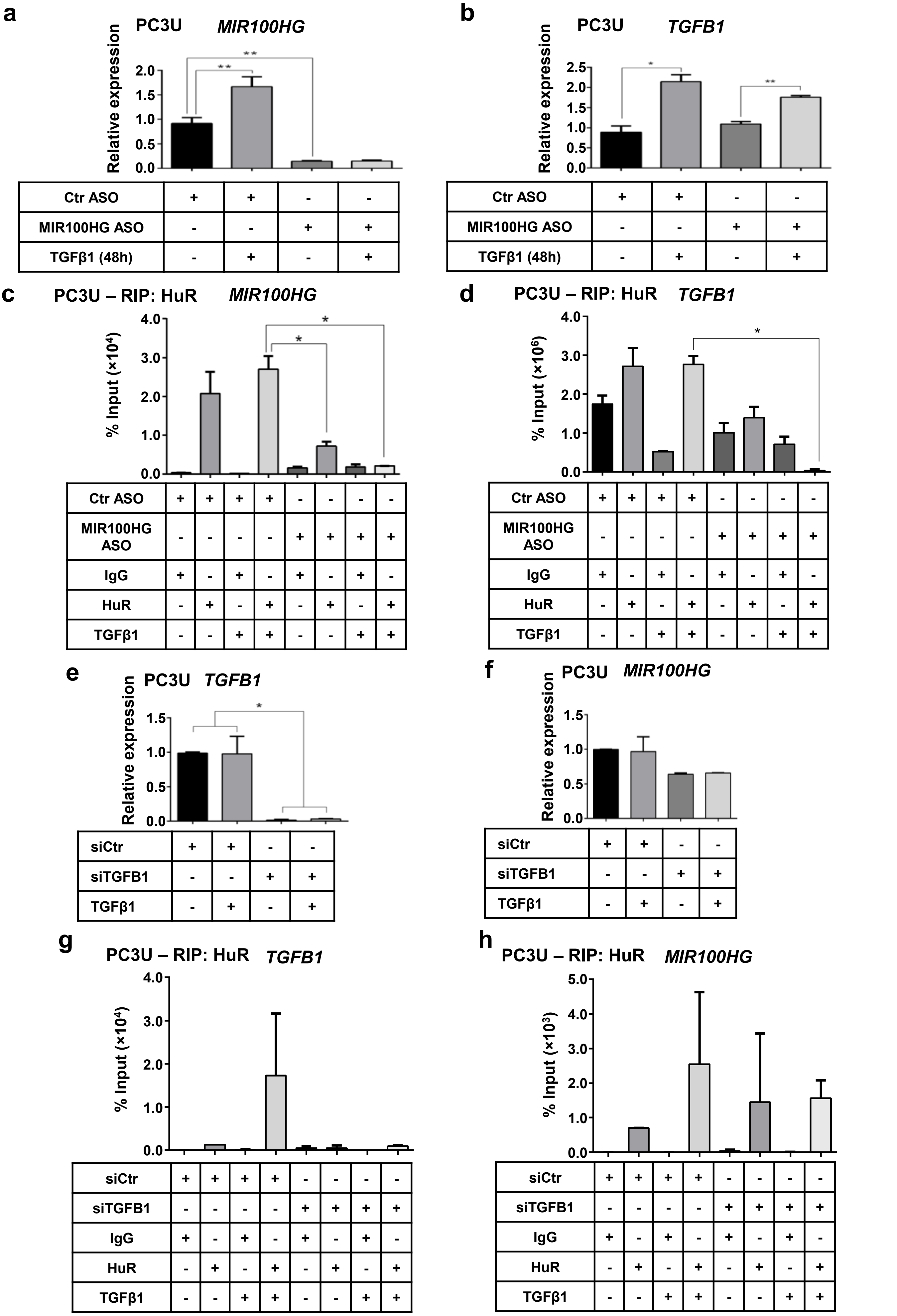
a
PC3U MIR100HG
Relative expression
Ctr ASO
MIR100HG ASO
TGFβ1 (48h)
b
PC3U TGFB1
Relative expression
Ctr ASO
MIR100HG ASO
TGFβ1 (48h)
c
PC3U – RIP: HuR MIR100HG
% Input (×10⁴)
Ctr ASO
MIR100HG ASO
IgG
HuR
TGFβ1
d
PC3U – RIP: HuR TGFB1
% Input (×10⁶)
Ctr ASO
MIR100HG ASO
IgG
HuR
TGFβ1
e
PC3U TGFB1
Relative expression
siCtr
siTGFB1
TGFβ1
f
PC3U MIR100HG
Relative expression
siCtr
siTGFB1
TGFβ1
g
PC3U – RIP: HuR TGFB1
% Input (×10⁴)
siCtr
siTGFB1
IgG
HuR
TGFβ1
h
PC3U – RIP: HuR MIR100HG
% Input (×10³)
siCtr
siTGFB1
IgG
HuR
TGFβ1

Figure S8

**Suppl. Fig. S8.** *MIR100HG* engages HuR to regulate TGFβ1. **a, b** Real time RT-qPCR for detection of *MIR100HG* (a) and *TGFB1* (b) expression in PC3U cells transiently transfected with negative control or anti-MIR100HG ASO and stimulated with TGFβ1 or not for 48 h. Gene expression is normalized relative to the housekeeping gene *HPRT1*. Error bars represent standard deviation from three different experiments (*$p$<0.05, **$p$<0.01). **c, d** RIP analysis in PC3U cells transiently transfected with negative control or anti-MIR100HG ASO and stimulated with TGFβ1 or not for 48 h. Percent of the HuR-specific RIP relative to the input RNA is reported for *MIR100HG* (c) and *TGFB1* (d) RNAs. RIP using non-specific IgG control antibody is also shown. Error bars represent standard deviation from three different experiments (*$p$<0.05). **e, f** Real time RT-qPCR for detection of *TGFB1* and *MIR100HG* expression in PC3U cells transiently transfected with negative control or *TGFB1*-specific siRNA#6 (Suppl. Table S2) and treated or not with TGFβ1 for 48 h. Gene expression is normalized relative to the housekeeping gene *HPRT1*. Error bars represent standard deviation from three different experiments (*$p$<0.05). **g, h** RIP analysis in PC3U cells transiently transfected with negative control or *TGFB1*-specific siRNA and stimulated with TGFβ1 or not for 48 h. Percent of the HuR-specific RIP relative to the input RNA is reported for *TGFB1* (g) and *MIR100HG* (h) RNAs. Lack of stars indicates lack of statistically significant differences.

HaCaT

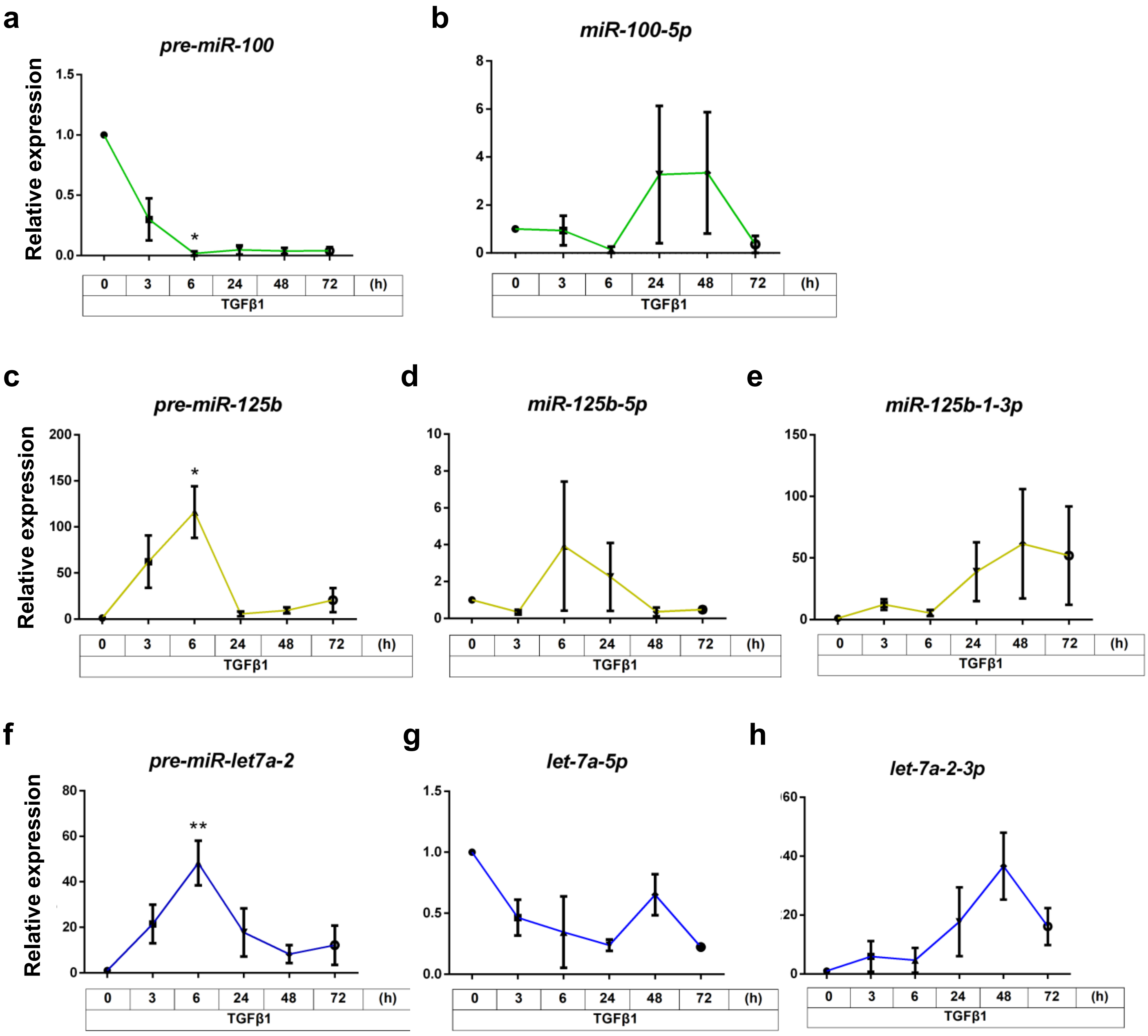


Figure S9

**Suppl. Figure S9.** Regulation of *MIR100HG* intron-3 miRNAs by TGFβ in HaCaT cells. **a-h** TaqMan real-time RT-qPCR assays to determine the expression of the indicated precursor (pre) and corresponding mature miRNAs in HaCaT cells treated with TGFβ1 for the indicated time periods. Gene expression is normalized relative to the housekeeping miRNA *miR-191-5p*. Error bars represent standard error of the mean from three different experiments (**p*<0.05, ***p*<0.01).

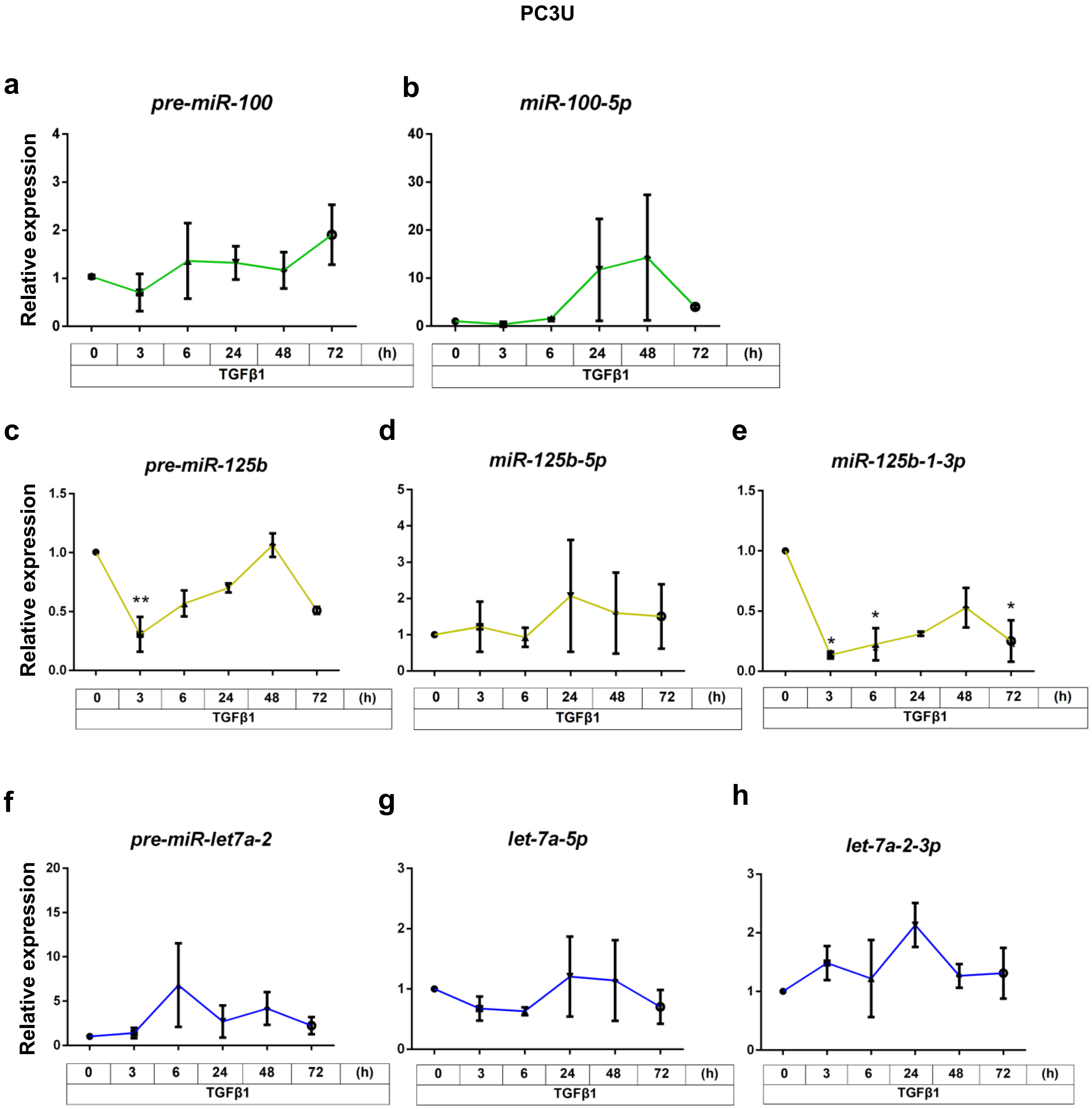

PC3U
a
pre-miR-100
Relative expression
b
miR-100-5p
c
pre-miR-125b
d
miR-125b-5p
e
miR-125b-1-3p
f
pre-miR-let7a-2
g
let-7a-5p
h
let-7a-2-3p
0
3
6
24
48
72
(h)
TGFβ1


Figure S10

**Suppl. Figure S10.** Regulation of *MIR100HG* intron-3 miRNAs by TGFβ in PC3U cells. **a**-**h** TaqMan real-time RT-qPCR assays to determine the expression of the indicated precursor (pre) and corresponding mature miRNAs in PC3U cells treated with TGFβ1 for the indicated time periods. Gene expression is normalized relative to the housekeeping miRNA *miR-191-5p*. Error bars represent standard error of the mean from three different experiments (**p*<0.05, ***p*<0.01).

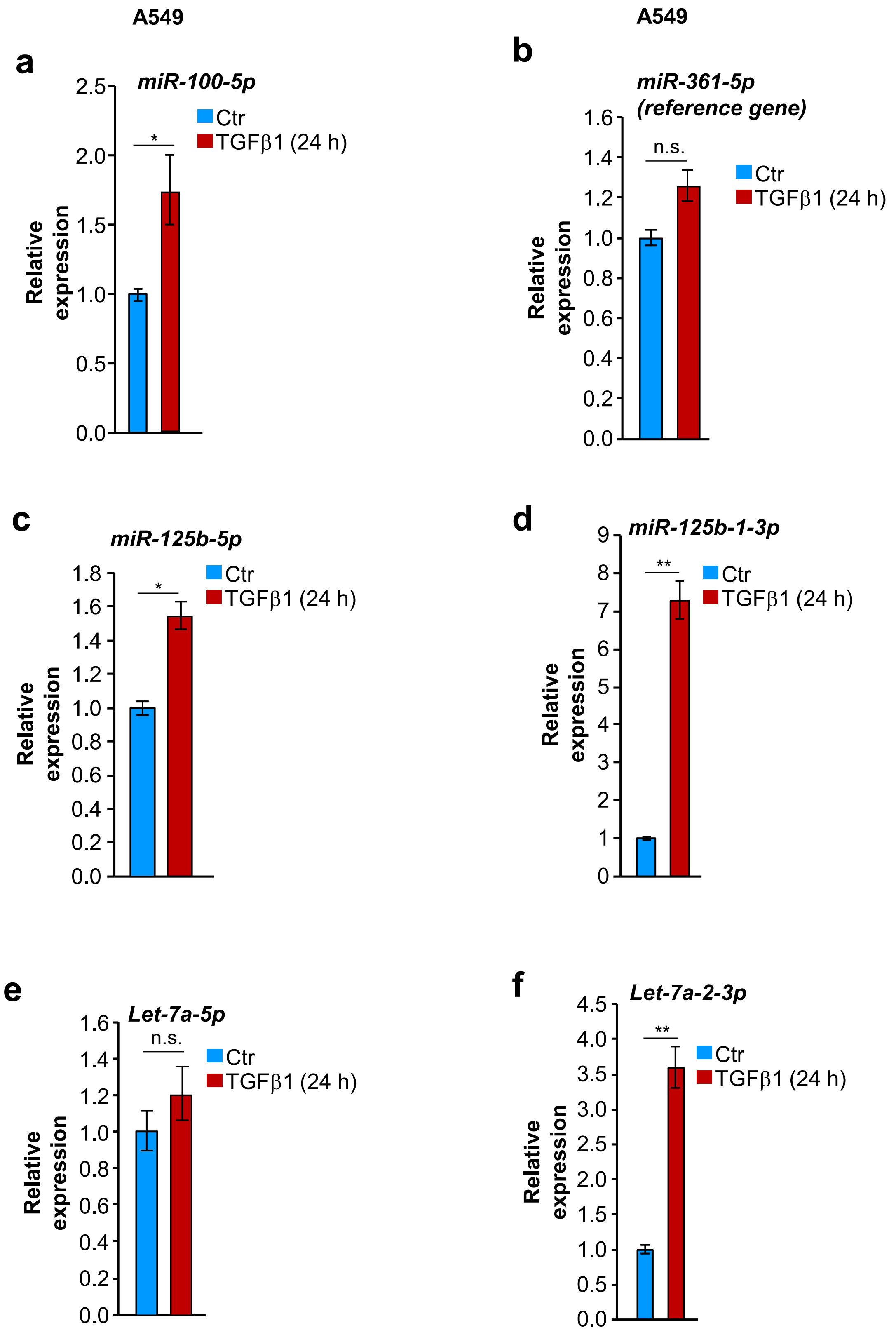

A549
A549
a
miR-100-5p
2.5
2.0
1.5
1.0
0.5
0.0
Relative expression
*
Ctr
TGFβ1 (24 h)
b
miR-361-5p (reference gene)
1.6
1.4
1.2
1.0
0.8
0.6
0.4
0.2
0.0
n.s.
Ctr
TGFβ1 (24 h)
Relative expression
c
miR-125b-5p
1.8
1.6
1.4
1.2
1.0
0.8
0.6
0.4
0.2
0.0
*
Ctr
TGFβ1 (24 h)
Relative expression
d
miR-125b-1-3p
9
8
7
6
5
4
3
2
1
0
**
Ctr
TGFβ1 (24 h)
Relative expression
e
Let-7a-5p
1.6
1.4
1.2
1.0
0.8
0.6
0.4
0.2
0.0
n.s.
Ctr
TGFβ1 (24 h)
Relative expression
f
Let-7a-2-3p
4.5
4.0
3.5
3.0
2.5
2.0
1.5
1.0
0.5
0.0
**
Ctr
TGFβ1 (24 h)
Relative expression


Figure S11

**Suppl. Figure S11.** Regulation of *MIR100HG* intron-3 miRNAs by TGFβ in A549 cells. **a-f** TaqMan real-time RT-qPCR assays to determine the expression of the indicated mature miRNAs in A549 cells treated with TGFβ1 for 24 h. Gene expression is normalized relative to the housekeeping miRNA *miR-191-5p*. Error bars represent standard deviation from three different experiments (**p*<0.05, ***p*<0.01, n.s.: not significant).

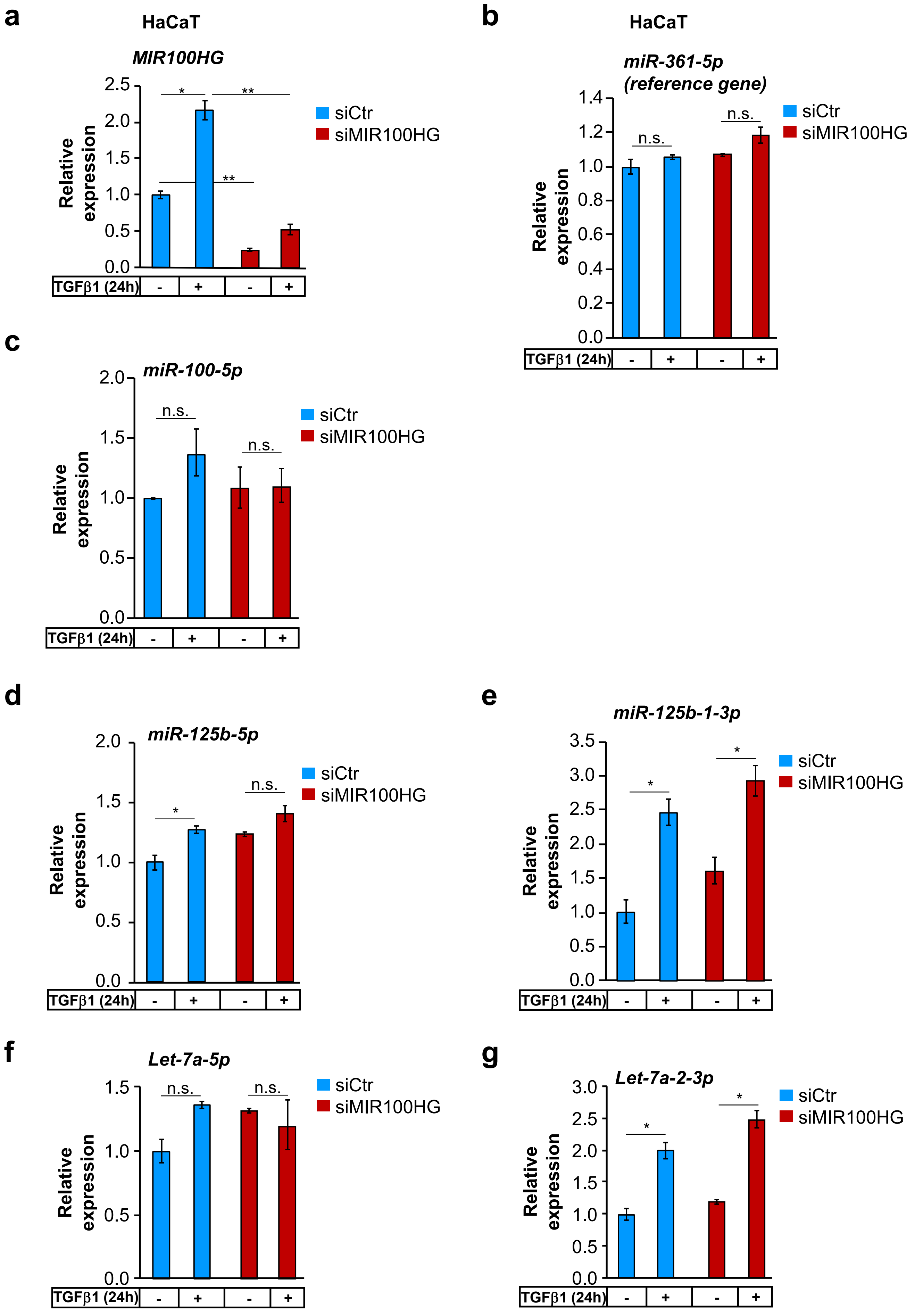
a
HaCaT
MIR100HG
Relative expression
2.5
2.0
1.5
1.0
0.5
0.0
*
**
**
siCtr
siMIR100HG
TGFβ1 (24h)
-
+
-
+
b
HaCaT
miR-361-5p
(reference gene)
1.4
1.2
1.0
0.8
0.6
0.4
0.2
0.0
n.s.
n.s.
c
miR-100-5p
2.0
1.5
1.0
0.5
0.0
d
miR-125b-5p
e
miR-125b-1-3p
3.5
3.0
f
Let-7a-5p
g
Let-7a-2-3p

Figure S12

**Suppl. Figure S12.** *MIR100HG*-derived miRNAs are expressed independently of the mature *MIR100HG*. **a** Real time RT-qPCR for detection of *MIR100HG* expression in HaCaT cells transiently transfected with the control of *MIR100HG*-specific siRNApool-of-4 and stimulated with TGFβ1 or not for 24 h. Gene expression is normalized relative to the housekeeping gene *18S rRNA*. Error bars represent standard deviation from three different experiments (**p*<0.05, ***p*<0.01). **b**-**g** TaqMan real-time RT-qPCR assays to determine the expression of the indicated mature miRNAs (*miR-361-5p* (b) served as reference gene for lack of responsiveness to TGFβ signaling), in the same HaCaT cells transfected as in (a) and treated with TGFβ1 or not for 24 h. Gene expression is normalized relative to the housekeeping miRNA *miR-191-5p*. Error bars represent standard deviation from three different experiments (**p*<0.05, n.s.: not significant).

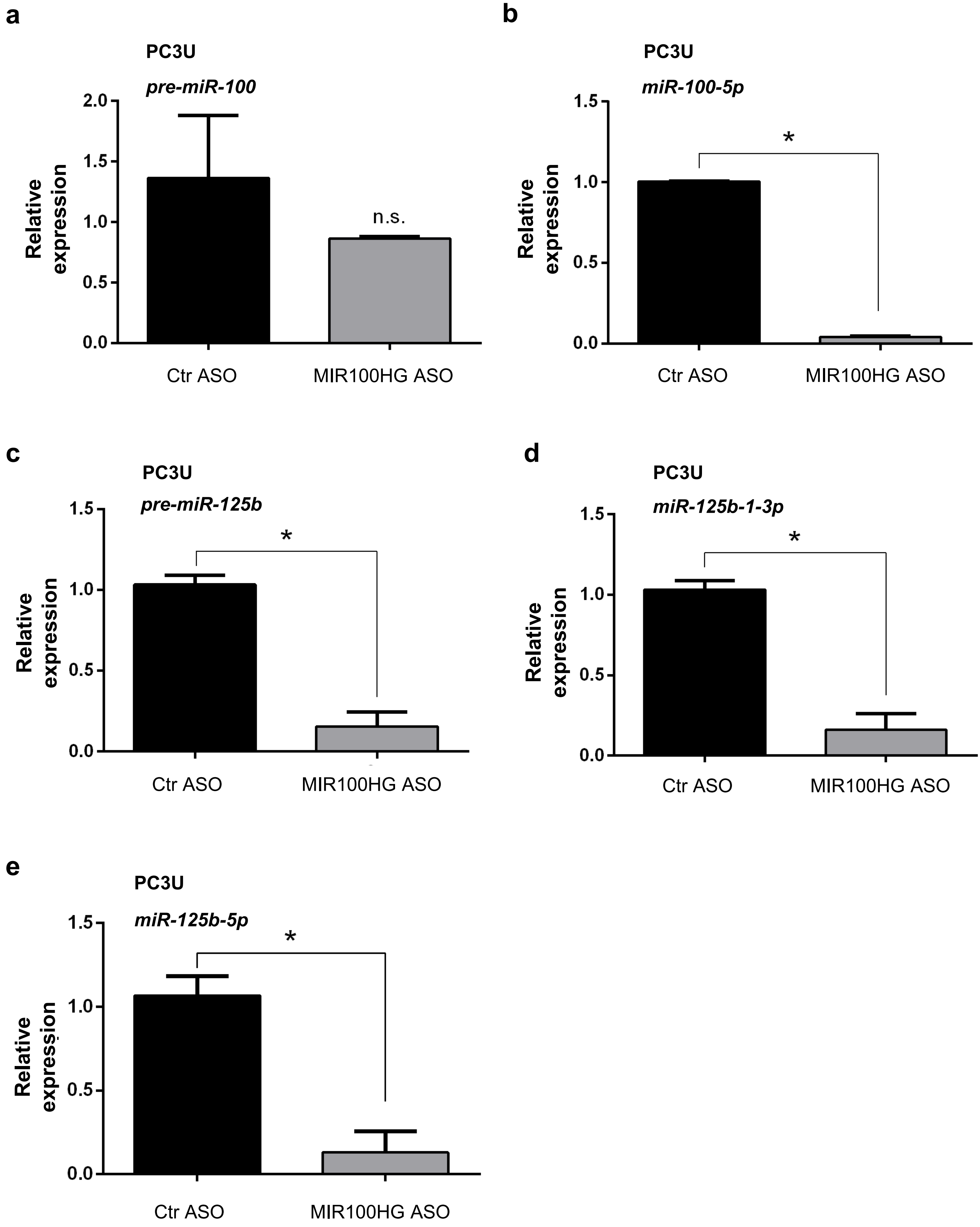
a
PC3U
pre-miR-100
Relative expression
2.0
1.5
1.0
0.5
0.0
n.s.
Ctr ASO
MIR100HG ASO
b
PC3U
miR-100-5p
Relative expression
1.5
1.0
0.5
0.0
*
Ctr ASO
MIR100HG ASO
c
PC3U
pre-miR-125b
Relative expression
1.5
1.0
0.5
0.0
*
Ctr ASO
MIR100HG ASO
d
PC3U
miR-125b-1-3p
Relative expression
1.5
1.0
0.5
0.0
*
Ctr ASO
MIR100HG ASO
e
PC3U
miR-125b-5p
Relative expression
1.5
1.0
0.5
0.0
*
Ctr ASO
MIR100HG ASO

Figure S13

**Suppl. Figure S13.** Impact of *MIR100HG* on *MIR100HG*-derived miRNA expression. **a-e** TaqMan real-time RT-qPCR assays to determine the expression of the indicated precursor (pre) and corresponding mature miRNAs in PC3U cells transiently transfected with negative control or anti-MIR100HG ASO in the absence of treatment with TGFβ1. Gene expression is normalized relative to the housekeeping miRNA *miR-191-5p*. Error bars represent standard deviation from three different experiments (*$p$<0.05, n.s.: not significant).

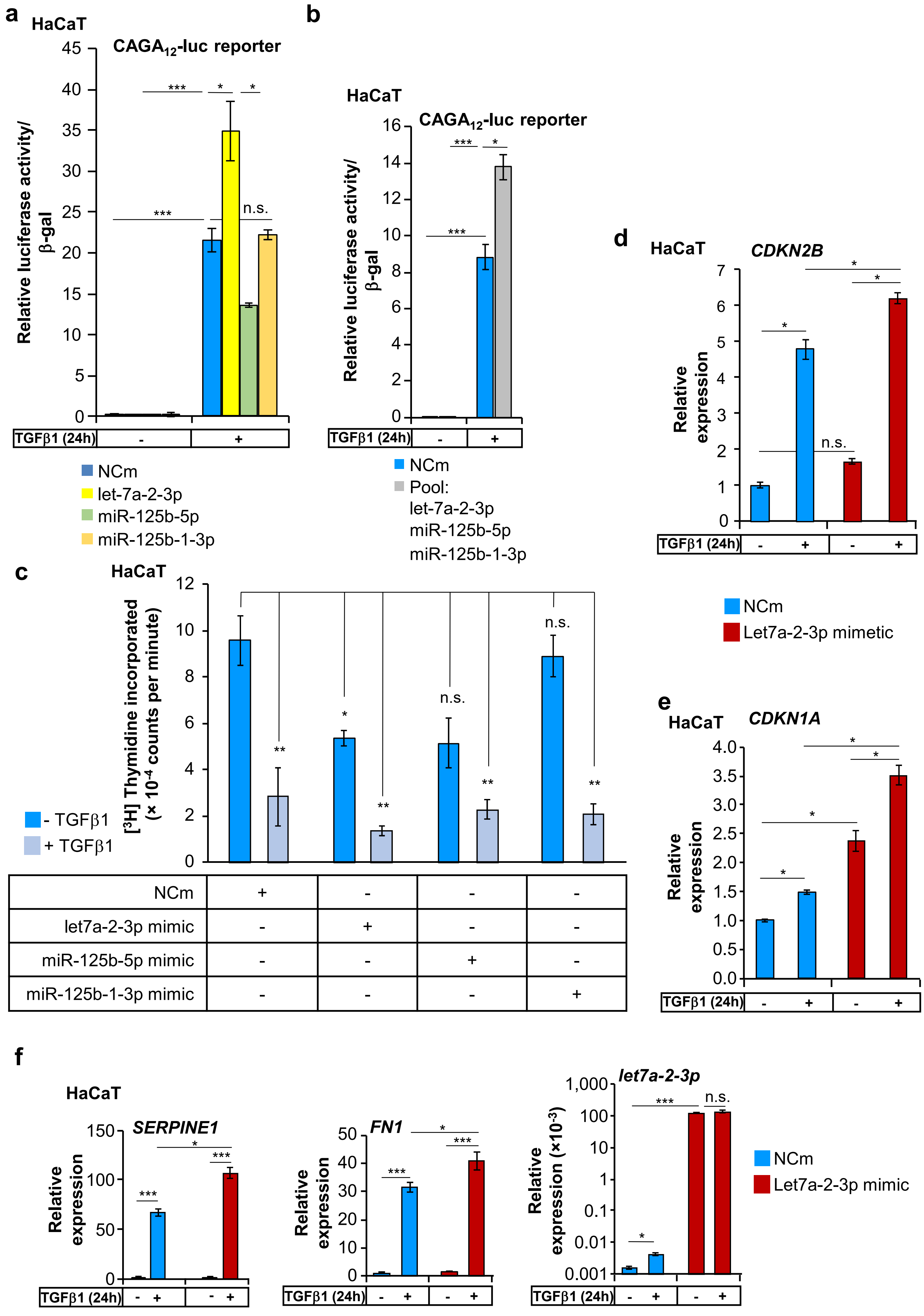

a
HaCaT
CAGA12-luc reporter
Relative luciferase activity/ β-gal
TGFβ1 (24h) - +
NCm
let-7a-2-3p
miR-125b-5p
miR-125b-1-3p
n.s.
b
HaCaT
CAGA12-luc reporter
Relative luciferase activity/ β-gal
TGFβ1 (24h) - +
NCm
Pool: let7a-2-3p miR-125b-5p miR-125b-1-3p
c
HaCaT
[3H] Thymidine incorporated (× 10-4 counts per minute)
- TGFβ1
+ TGFβ1
n.s.
NCm + - - -
let7a-2-3p mimic - + - -
miR-125b-5p mimic - - + -
miR-125b-1-3p mimic - - - +
d
HaCaT
CDKN2B
Relative expression
TGFβ1 (24h) - + - +
NCm
Let7a-2-3p mimetic
e
HaCaT
CDKN1A
Relative expression
TGFβ1 (24h) - + - +
f
HaCaT
SERPINE1
FN1
let7a-2-3p
Relative expression
Relative expression (×10-3)
TGFβ1 (24h) - + - +
NCm
Let7a-2-3p mimic


Figure S14

**Suppl. Figure S14.** The *let-7a-2-3p* miRNA positively regulates TGFβ signaling and promotes cell growth arrest. **a** $CAGA_{12}$-luciferase assay in HaCaT cells transiently overexpressing *let-7a-2-3p*, *miR-125b-5p* or *miR-125b-1-3p* miRNA-mimics or control non-coding miRNA (NCm), and stimulated with or without TGFβ1 for 24 h. Error bars represent standard deviation from three different experiments (**p*<0.05, ****p*<0.001, n.s., not significant). **b** $CAGA_{12}$-luciferase assay in HaCaT cells transiently transfected and simultaneously overexpressing the three miRNA-mimics *let-7a-2-3p*, *miR-125b-5p* and *miR-125b-1-3p* or control non-coding miRNA (NCm), and stimulated or not with TGFβ1 for 24 h. Error bars represent standard deviation from three different experiments (**p*<0.05, ****p*<0.001, n.s., not significant). **c** $^{3}$H-thymidine incorporation assay in HaCaT cells transiently overexpressing *let-7a-2-3p*, *miR-125b-5p*, *miR-125b-1-3p* miRNA-mimics or non-specific control miRNA (NCm) and treated or not with TGFβ1 for 24 h. Error bars represent standard deviation from three different experiments (**p*<0.05, ***p*<0.01, n.s., not significant). **d**, **e** Real time RT-qPCR for determination of *CDKN2B* (d) or *CDKN1A* (e) mRNA in HaCaT cells transiently overexpressing *let-7a-2-3p*-mimic and treated or not with TGFβ1 for 24 h. Gene expression is normalized relative to the housekeeping gene *18S rRNA*. Error bars represent standard deviation from three different experiments (**p*<0.05, n.s.: not significant). **f** Real time RT-qPCR for determination of *SERPINE1 and FN1* and TaqMan assay to measure mature *let-7a-2-3p* expression in HaCaT cells transiently transfected with *let-7a-2-3p* miRNA-mimic or the corresponding negative control mimics (NCm), after treatment with TGFβ1 or not for 24 h. *GAPDH* was used as a reference gene for normalization of mRNAs and *miR-191-5p* as a reference gene for normalizing *let-7a-2-3p* levels. The values for the expression of *let-7a-2-3p* are shown

in logarithmic scale. Error bars represent standard deviation from three different experiments (**p*<0.05, ****p*<0.001, n.s., not significant).

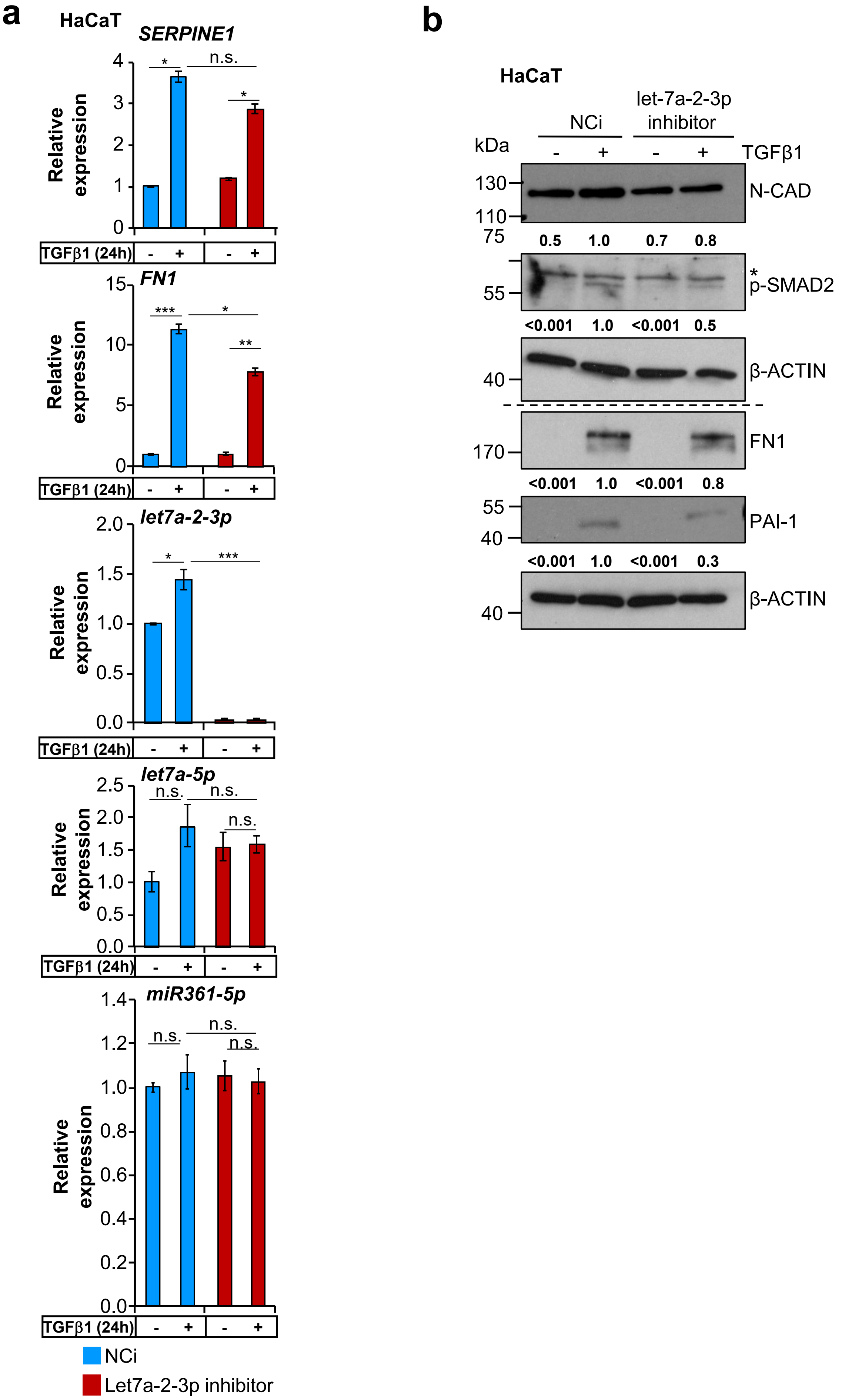

a
HaCaT
SERPINE1
Relative expression
n.s.
TGFβ1 (24h)
FN1
let7a-2-3p
let7a-5p
miR361-5p
NCi
Let7a-2-3p inhibitor
b
HaCaT
NCi
let-7a-2-3p inhibitor
kDa
TGFβ1
N-CAD
0.5 1.0 0.7 0.8
p-SMAD2
<0.001 1.0 <0.001 0.5
β-ACTIN
FN1
<0.001 1.0 <0.001 0.8
PAI-1
<0.001 1.0 <0.001 0.3
β-ACTIN


Figure S15

**Suppl. Figure S15.** The *let-7a-2-3p* miRNA regulates TGFβ signaling. **a** Real time RT-qPCR for determination of *SERPINE1 and FN1* and TaqMan assay to measure *let-7a-2-3p* expression in HaCaT cells transiently transfected with *let-7a-2-3p* miRNA-inhibitor or the corresponding negative control inhibitor (NCi) and after treatment or not with TGFβ1 for 24 h. TaqMan assays for *let-7a-5p* (specificity control) and *miR361-5p* (reference miRNA) are also included. MiRNA expression is normalized relative to the housekeeping miRNA *miR-191-5p*, which is not regulated by TGFβ. Error bars represent standard deviation from three different experiments (*$p<0.05$, **$p<0.01$, ***$p<0.001$, n.s., not significant). **b** Representative immunoblots of N-CAD and phosphorylated SMAD2 (p-SMAD2) (top immunoblot) and FN1 and PAI-1 (bottom immunoblot) in HaCaT cells transiently transfected with *let-7a-2-3p* miRNA-inhibitor or the corresponding negative control inhibitor (NCi) and after treatment or not with TGFβ1 for 24 h. β-ACTIN was used as a loading control. Molecular mass (kDa) markers are indicated along with densitometric values of normalized band intensity. A star indicates a non-specific protein band recognized by the antibody.

**a**

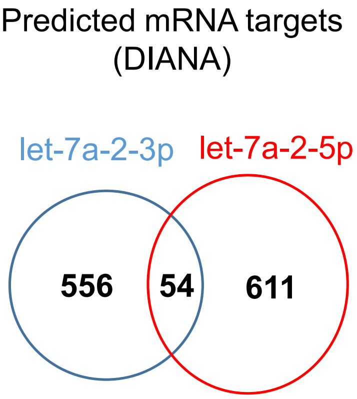


**b**

| mirPath v.3 (DIANA) | | | | |
|---|---|---|---|---|
| | Name | *p*-value | Number of genes | Gene symbol |
| **Let-7a-2-3p targets** | **Glycosaminoglycan biosynthesis - heparan sulfate / heparin** | **$1.61E^{-07}$** | **5** | **EXT1, HS3ST1, HS3ST3B1, GLCE, NDST3** |
| | **Wnt signaling pathway** | **0.000586** | **16** | **FZD7, LRP6, VANGL1,TCF7L2, CUL1, PPP3CA, NLK, PLCB1, FZD4, SOST, GPC4, VANGL2, RAC1, CXXC4, DAAM1, TBL1XR1** |
| | **Lysine methyltransferase** | **0.000731** | **5** | **KMT2D, SUV420H1, KMT2E, KMT2C, SETD1A** |

**c**

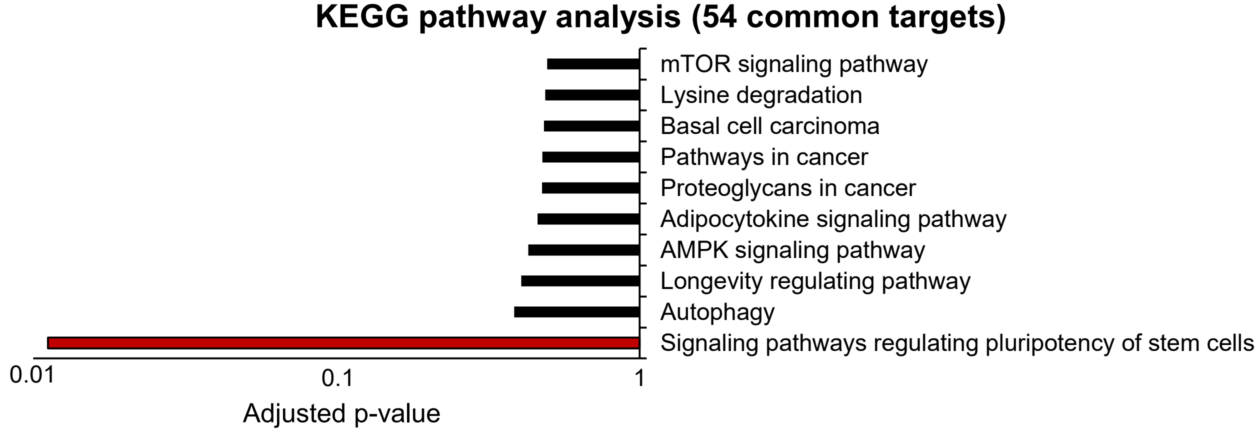


**d**

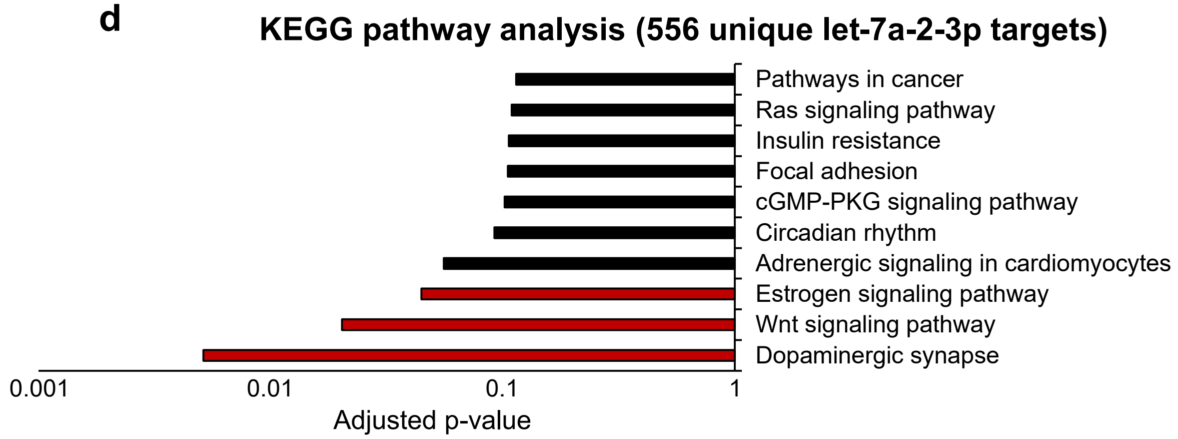


**e**

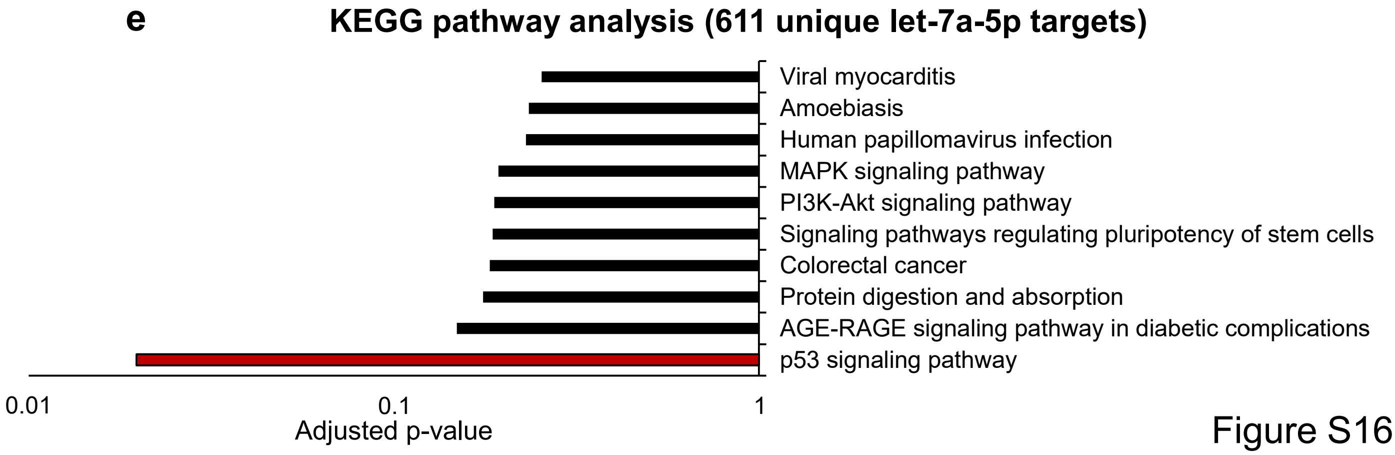


Figure S16

**Suppl. Figure S16.** *Let-7a-2-3p* target mRNAs. **a** DIANA tool analysis of *let-7a-2-3p* and *let-7a-2-5p* target mRNAs presented in Venn diagrams. **b** DIANA tool analysis of the 556 unique *let-7a-2-3p* target mRNAs reporting the top three functional categories of mRNAs with pathway name, corresponding *p*-value, total number of genes (mRNAs) included in each functional group and corresponding gene symbols. **c-e** KEGG pathway analysis of *let-7a-2-3p* and *let-7a-2-5p* common (54) target mRNAs (c), *let-7a-2-3p* unique (556) target mRNAs (d) and *let-7a-2-5p* unique (611) target mRNAs (e). Each graph presents functional categories along with associated adjusted *p*-value.

**a**

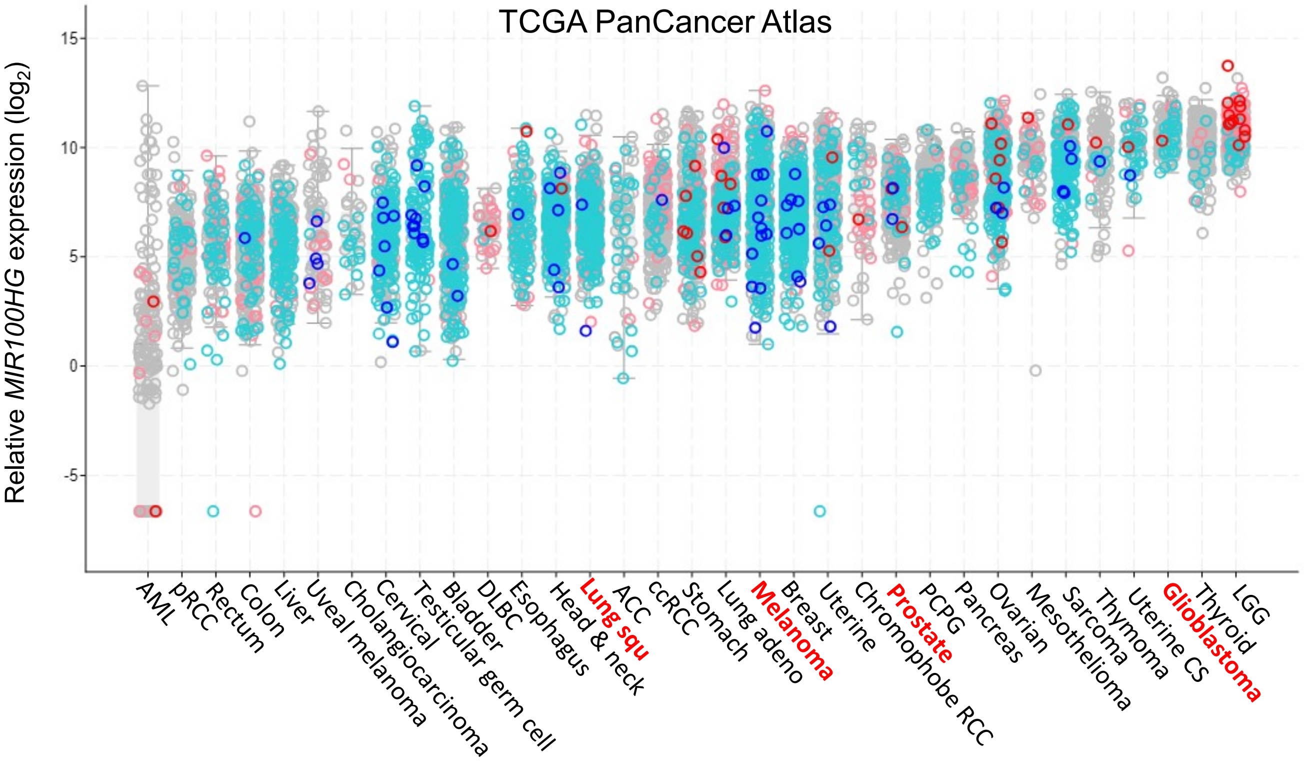


**b**

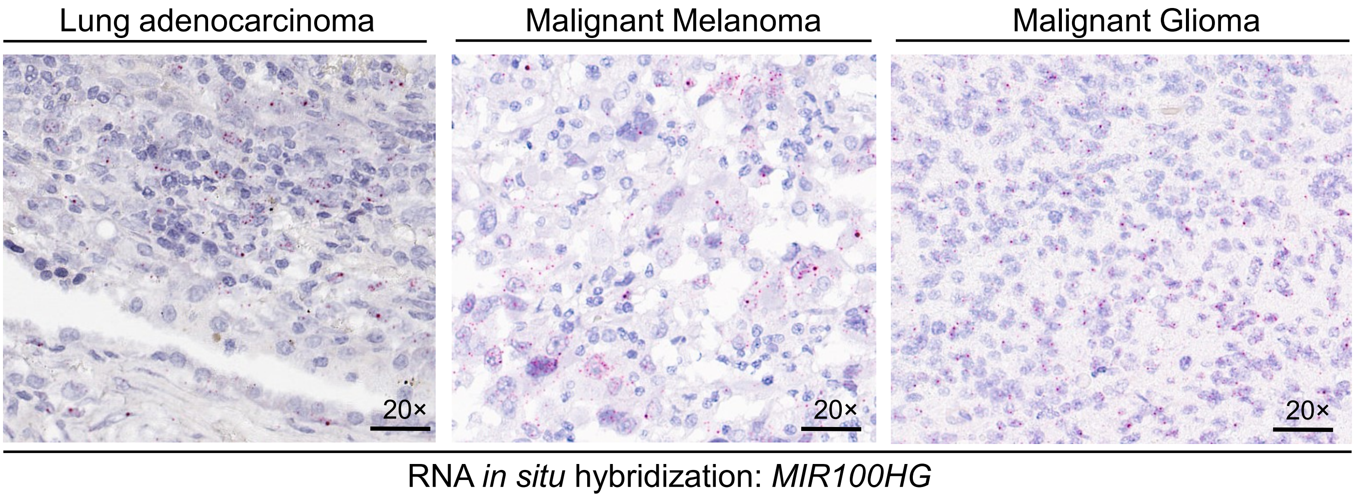


Figure S17

**Suppl. Figure S17.** *MIR100HG* expression in cancer patient samples. **a** *MIR100HG* RNA expression levels (normal logarithmic scale) in all of the cancer samples of the PanCancer Atlas of TCGA. Tumor types shown in red are those that have been analyzed further via RNA FISH or Kaplan-Meier plots. **b** In situ hybridization assay, using RNAscope for detection of *MIR100HG* expression in lung, melanoma and glioma tumor tissues from patients. Representative pictures are shown along with magnification bar.

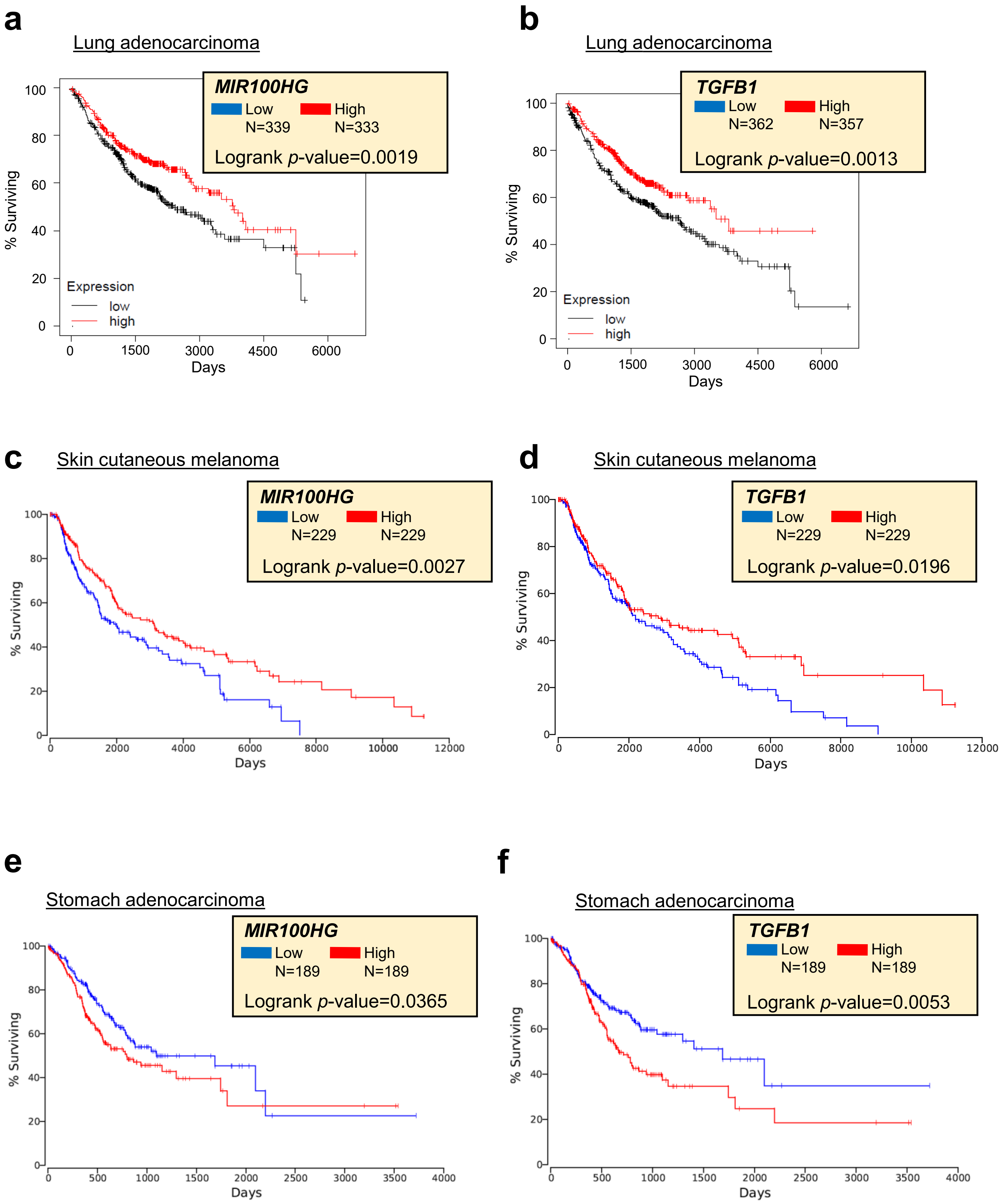

a
Lung adenocarcinoma
MIR100HG
Low
N=339
High
N=333
Logrank p-value=0.0019
% Surviving
Days
Expression
low
high
b
Lung adenocarcinoma
TGFB1
Low
N=362
High
N=357
Logrank p-value=0.0013
% Surviving
Days
Expression
low
high
c
Skin cutaneous melanoma
MIR100HG
Low
N=229
High
N=229
Logrank p-value=0.0027
% Surviving
Days
d
Skin cutaneous melanoma
TGFB1
Low
N=229
High
N=229
Logrank p-value=0.0196
% Surviving
Days
e
Stomach adenocarcinoma
MIR100HG
Low
N=189
High
N=189
Logrank p-value=0.0365
% Surviving
Days
f
Stomach adenocarcinoma
TGFB1
Low
N=189
High
N=189
Logrank p-value=0.0053
% Surviving
Days


Figure S18

**Suppl. Figure S18.** Correlation of *MIR100HG* expression and survival outcome in cancer patients. Kaplan-Meier survival curves for *MIR100HG* (**a**, **c**, **e**) and *TGFB1* (**b**, **d**, **f**) in lung adenocarcinoma (a, b), cutaneous melanoma (c, d) and stomach adenocarcinoma (e, f) patients. The number of patient samples included in the analysis along with the logrank *p*-value is indicated.
